# A Cyanine‐Bridged Somatostatin Hybrid Probe for Multimodal SSTR2 Imaging in Vitro and in Vivo: Synthesis and Evaluation

**DOI:** 10.1002/cbic.202000791

**Published:** 2021-01-04

**Authors:** Isabelle Heing‐Becker, Carsten Grötzinger, Nicola Beindorff, Sonal Prasad, Sarah Erdmann, Samantha Exner, Rainer Haag, Kai Licha

**Affiliations:** ^1^ Institut für Chemie und Biochemie Freie Universität Berlin Takustr. 3 14195 Berlin Germany; ^2^ Department of Hepatology and Gastroenterology Charité – Universitätsmedizin Berlin Augustenburger Platz 1 13353 Berlin Germany; ^3^ BERIC – Berlin Experimental Radionuclide Imaging Center Charité – Universitätsmedizin Berlin Augustenburger Platz 1 13353 Berlin Germany; ^4^ Department of Nuclear Medicine Charité – Universitätsmedizin Berlin Augustenburger Platz 1 13353 Berlin Germany

**Keywords:** cyanines, fluorescence, multimodal imaging, octreotate, SSTR2

## Abstract

Multimodal imaging probes have attracted the interest of ongoing research, for example, for the surgical removal of tumors. Modular synthesis approaches allow the construction of hybrid probes consisting of a radiotracer, a fluorophore and a targeting unit. We present the synthesis of a new asymmetric bifunctional cyanine dye that can be used as a structural and functional linker for the construction of such hybrid probes. ^68^Ga‐DOTATATE, a well‐characterized radiopeptide targeting the overexpressed somatostatin receptor subtype 2 (SSTR2) in neuroendocrine tumors, was labeled with our cyanine dye, thus providing additional information along with the data obtained from the radiotracer. We tested the SSTR2‐targeting and imaging properties of the resulting probe ^68^Ga‐DOTA‐ICC‐TATE *in vitro* and in a tumor xenograft mouse model. Despite the close proximity between dye and pharmacophore, we observed a high binding affinity towards SSTR2 as well as elevated uptake in SSTR2‐overexpressing tumors in the positron emission tomography (PET) scan and histological examination.

## Graphical abstract

**Multifunctional cyanine dyes**: Multimodal imaging agents can be built up based on an asymmetric cyanine dye connecting the radionuclide and targeting unit. Insertion of a cyanine dye into the clinically approved radiopharmaceutical ^68^Ga‐DOTATATE allowed the identification of cancer tissue *in vivo* and *ex vivo* by using radionuclear and fluorescence imaging.

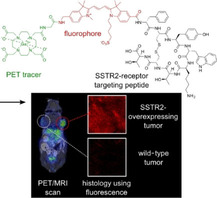

## Introduction

Multimodal imaging using PET or SPECT (single photon emission computed tomography) together with fluorescence imaging has emerged as a promising tool for the development of new diagnostics and treatment options.^[1]^ Instead of using different imaging agents for different imaging modalities in various experimental setups, only one probe is used for all imaging procedures. The evident advantages for preclinical and clinical applications include the complementary data obtained from both modalities having different sensitivities, resolutions and penetration depths along with a reduction of time, costs and resources needed.^[2]^

A major application of hybrid probes lies in the translation into image‐guided oncologic surgery.^[3]^ The different modalities help the surgeon to first localize the tumor via the PET or SPECT scan, followed by the identification of tumor margins through fluorescence imaging, which enables the resection of affected areas.^[4]^ Beyond intraoperative imaging, hybrid probes are also interesting for the evaluation of new drug candidates because the same label can be used for subsequent *in vitro, in vivo* and *ex vivo* studies. In this way, difficulties in data interpretation resulting from different labels with different biological behavior are circumvented.

For these purposes, labels consisting of a fluorophore being either attached to a chelator for radionuclide complexation^[5]^ or subjected to ^18^F‐fluorination^[6]^ have been developed and conjugated to specific targeting molecules such as small molecules,^[7]^ peptides,^[8]^ antibodies^[9]^ or polymers.^[10]^ When building up hybrid probes, the question arises of how to position the three components radiotracer, fluorophore and targeting unit. This aspect is mainly dictated by the availability of free conjugatable sites of the components. The vast majority of hybrid probes either contain an extra linking platform (based, e. g., on lysine^[11]^ or cyclooctyne^[12]^) or use the radionuclide chelator as the linker between fluorophore and targeting unit.^[3]^

In our study, we present a new design approach for the construction of multimodal imaging probes and employ the fluorophore as the linking unit between chelator and targeting moiety. Among the different classes of fluorescent dyes, cyanine dyes possess a high structural modifiability that can be used to introduce various functional groups.^[13]^ We exploited this aspect to design and synthesize a new asymmetric bifunctional indocarbocyanine (ICC) dye which can serve as a linker between two heterofunctional groups.

To exemplify the usage of this new ICC dye, we decided to focus on the radiopeptide ^68^Ga‐DOTATATE that has attracted particular interest for the construction of multimodal imaging probes.[^3^, ^14^] As somatostatin analogs, the peptides Phe1‐Tyr3‐octreotate (TATE) and the closely related Phe1‐Tyr3‐octreotide (TOC) target the somatostatin receptor (SSTR) subtype 2, which can be overexpressed in neuroendocrine tumors (NETs).^[15]^ Their radiolabeled forms ^68^Ga‐DOTATATE and ^68^Ga‐DOTATOC are the workhorses in the diagnosis of NETs and represent well‐characterized, clinically used radiodiagnostics for PET imaging of SSTR2.^[16]^ In these compounds, the DOTA chelator is connected via the N‐terminal amine of the peptide, leaving only the remaining functional groups of the peptide for the attachment of a fluorophore. However, Edwards et al. found that further functionalization of the Lys‐residue in TATE with a cyanine dye led to a poor *in vivo* tumor uptake and indicates that functionalization of certain residues in TATE can significantly diminish the peptide functionality towards SSTR2.^[17]^ In other approaches for multimodal SSTR2‐imaging, Ghosh et al. used a chelator with two binding sites as the linker for a cyanine dye and the peptide TOC as well as a linking platform to connect all three components.^[18]^ In this case, the dye was intentionally positioned far away from the peptide to minimize interactions.

Our study was conceived to investigate whether SSTR2‐targeted imaging is possible when using our bifunctional cyanine dye as the linker for the DOTA chelator and the targeting peptide TATE, with the dye being placed directly next to the pharmacophore. The resulting Ga‐DOTA‐ICC‐TATE probe was examined in cell experiments and in an SSTR2‐overexpressing tumor mouse model to evaluate its targeting and imaging properties on the *in vitro, in vivo* and *ex vivo* level.

## Results and Discussion

### Synthesis

**Scheme 1:**
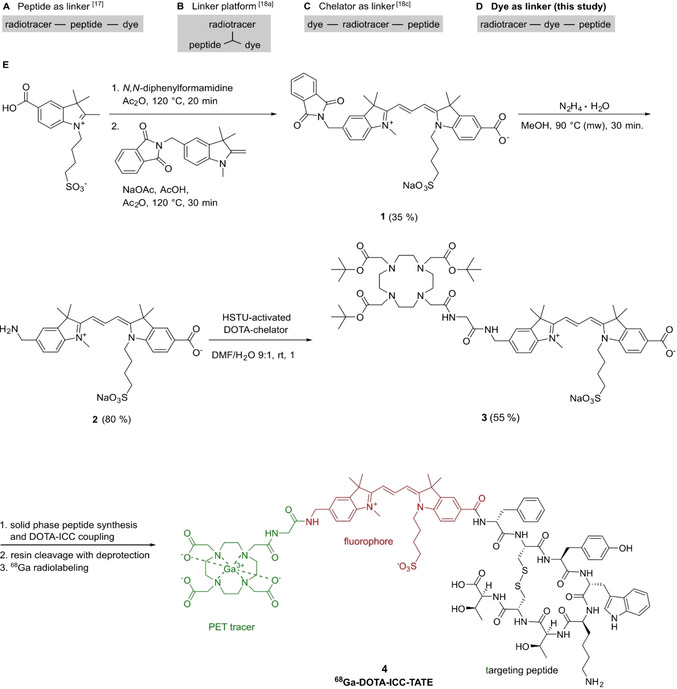
Different design approaches (A–C) for SSTR2‐targeting hybrid probes using either the peptide, the chelator or a platform as the linking structure. D) In this study, the dye is used as the linker. E) Synthesis of the new asymmetric bifunctional indocarbocyanine dye **2** and DOTA‐ICC label **3** as well as the chemical structure of the ^68^Ga‐DOTA‐ICC‐TATE probe **4** with the cyanine dye bridging the DOTA‐chelator and the peptide TATE.

Different strategies for the molecular design of somatostatin‐based hybrid probes have been developed (Scheme 1A–C) using the targeting peptide, the chelator or a dedicated component as the linker. To the best of our knowledge, employing the fluorophore as the linker for SSTR2‐targeting hybrid probes has not been done so far. We herein present a synthetic approach that opens up new possibilities for the construction of multimodal imaging probes. We used this strategy to create a dye‐linked hybrid probe for multimodal SSTR2‐imaging (Scheme 1D). To this end, we selected the clinically used radiotracer ^68^Ga‐DOTATATE and inserted a cyanine dye between the chelator and peptide. This molecular design was achieved by synthesizing an indocarbocyanine dye carrying two different conjugatable functional groups at the aromatic moieties. Whereas the functionalization of the indolic N‐substituents is widely known and applied,^[19]^ our design approach leaves these N‐substituents in principle open to modifications so that physicochemical properties like solubility or aggregation behavior can be adjusted.

We synthesized the asymmetric ICC dye **1** from a phthalimide functionalized Fischer's base, *N,N*‐diphenylformamidine and a carboxylic acid containing indolenine precursor, leading to a good yield of 35 % in terms of asymmetric dye synthesis (Scheme 1E). Hydrazinolysis according to the Ing‐Manske procedure in Gabriel's synthesis using hydrazine monohydrate in methanol yielded the new bifunctional ICC dye **2**. For the upscaling of this step, it was crucial to use a microwave setup for the heating, short heating times and repeated addition of hydrazine monohydrate in order to obtain good yields of up to 80 %. Performing the hydrazinolysis with the indodicarbocyanine (IDCC) analog led to a decrease of the yield, unfortunately, because longer polymethine chains are more prone towards decomposition by nucleophilic attacks. The water‐soluble bifunctional ICC dye **2** represents a unique linker platform for various applications, with a carboxylic acid on the one hand and an aliphatic amine that allows reactions with electrophiles under mild conditions on the other hand.

The amine functionality of this dye was used to attach a *tert*‐butyl‐protected, HSTU‐activated DOTA chelator to obtain the DOTA‐ICC label **3** in a yield of 55 % (Scheme 1E). For PET or SPECT studies, the DOTA chelator can in general form stable complexes with ^64^Cu, ^68^Ga and ^111^In.^[20]^ The methylene unit between the aromatic moiety and the amine of the dye was essential for the amide coupling. Without this additional methylene unit, harsh conditions were required being incompatible with acid‐labile protecting groups.

Afterwards, conventional coupling of the N‐terminus of TATE to the carboxyl group of the fluorophore was performed, followed by the removal of the remaining protective groups. In comparison to other design approaches, the resulting hybrid probe becomes relatively compact with the dye in direct proximity to the pharmacophore.

### Spectroscopic properties

The new bifunctional ICC dye **2** has a high absorption coefficient of 130 000 L mol^−1^ cm^−1^ in water with an absorption maximum at 551 nm and an emission maximum at 570 nm (Figure S1). For the ^nat^Ga‐DOTA‐ICC label, the absorption coefficient amounts to a good value of 80 000 L mol^−1^ cm^−1^ with an absorption maximum at 552 nm and an emission maximum at 570 nm. No significant changes in the fluorescence behavior compared to the free dye **2** were observed.

### *In vitro* studies

Toxicity assays demonstrated that the conjugate DOTA‐ICC‐TATE does not inhibit either metabolism or proliferation of human cells, up to a concentration of 10 μM (Figure S2).

**Figure 1:**
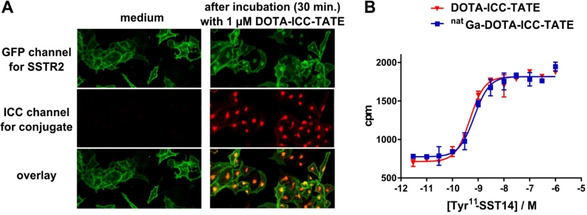
*In vitro* assessment of the hybrid probe. A) Cellular uptake studies with RIN1038 insulinoma cells overexpressing GFP‐fused SSTR2 at their surface (green). The cells were incubated in medium alone (left) or medium plus 1 μM DOTA‐ICC‐TATE (red, right). B) Concentration‐dependent displacement of the radioligand Tyr^11^‐SST14 by DOTA‐ICC‐TATE and ^nat^Ga‐DOTA‐ICC‐TATE.

The cellular uptake of the DOTA‐ICC‐TATE conjugate and the internalization of SSTR2 were analyzed. Therefore, RIN1038 cells overexpressing an SSTR2‐GFP fusion protein were used, allowing direct detection via GFP fluorescence. After incubation of the cells with the conjugate (1 μM) for 30 minutes, the strong fluorescent signal arising from the ICC dye could be clearly colocalized with the internalized vesicle‐associated GFP‐fused SSTR2 (Figure 1A), thus indicating the retention of the receptor‐binding and endocytosis‐stimulating properties of the peptide *in vitro*. According to the quantitative receptor internalization assay, DOTA‐ICC‐TATE stimulates the internalization of the SSTR2‐GFP fusion protein in a concentration‐dependent manner at a sub‐nanomolar EC_50_ value of 0.42 nM (Figure S2). The binding affinity towards SSTR2 was further supported by a competitive radioligand binding assay using ^125^I‐labeled Tyr^11^‐somatostatin‐14 as a tracer in BON‐SSTR2 cells (Figure 1B), yielding similar and low IC_50_ values of 0.77±0.3 nM for DOTA‐ICC‐TATE and 0.93±0.3 nM for ^nat^Ga‐DOTA‐ICC‐TATE. These values are in the range of published values for DOTATATE and Ga‐DOTATATE,^[21]^ thus suggesting that the close vicinity of the cyanine dye to the peptide TATE does not substantially influence the peptide functionality *in vitro*.

### *In vivo* imaging and biodistribution

Radiolabeling of the DOTA‐ICC‐TATE conjugate with ^68^Ga was performed at 95 °C over 500 s. Purification yielded the ^68^Ga‐DOTA‐ICC‐TATE probe **4** (Scheme 1E) in high radiochemical purity (>95 %) with a molar activity of 40 GBq/μmol.

**Figure 2:**
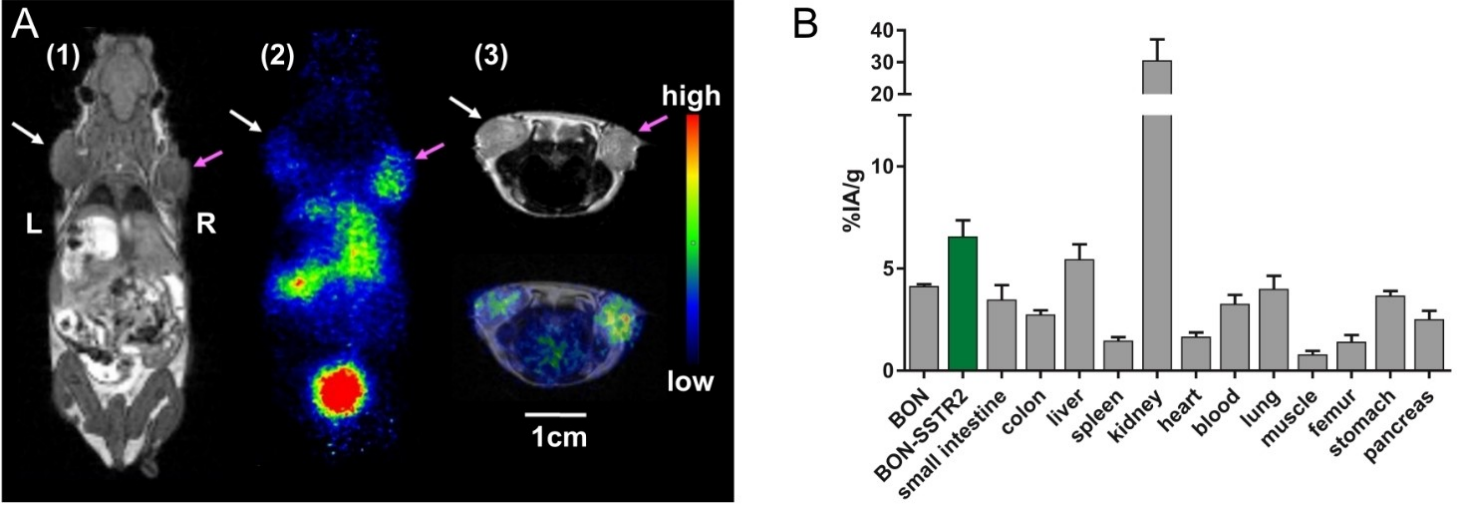
A) Illustrative PET/MRI images of an SSTR2‐overexpressing BON‐tumor‐bearing living mouse, acquired 41 min after tail vein injection of ^68^Ga‐DOTA‐ICC‐TATE. Pink arrows indicate the SSTR2‐overexpressing BON tumor, white arrows indicate the wild‐type BON tumor. The scale bar is representative for all images. 1) T1 coronal MRI image; 2) PET coronal image; 3) T2 FSE 2D transverse MRI and fusion with PET image. B) *Ex vivo* biodistribution data in xenograft‐bearing nude mice (*n*=3) 1 h after injection of ^68^Ga‐DOTA‐ICC‐TATE.

*In vivo* PET imaging was performed 41–48 minutes after injection of 8.8–14.3 MBq (0.2–0.3 nmol) of the ^68^Ga‐DOTA‐ICC‐TATE probe into nude mice bearing an SSTR2‐overexpressing tumor on the right and a wild‐type tumor on the left shoulder (Figures S3–S5). As shown in Figure 2A, the tumor could be well visualized by the PET/MRI scan. The uptake of the probe in the SSTR2‐overexpressing tumor was higher than in the wild‐type tumor with average standard uptake values of 6.6±1.3 % IA mL^−1^ in the SSTR2‐overexpressing tumor and uptake values of 4.6±0.3 % IA mL^−1^ in the wild‐type tumors. Substantial uptake of the probe was also seen in the kidneys and the bladder, but generally low in other organs.

*Ex vivo* biodistribution data were obtained 1 h after injection in the same tumor mouse model (Figure 2B). The data is in good agreement with the PET/MRI scans with uptake values of 6.7±1.4 % IA g^−1^ in the SSTR2‐overexpressing tumor, being higher than in the wild‐type tumor (4.1±0.2 % IA g^−1^). In other organs, a high accumulation was found in the kidneys (∼30 % IA g^−1^) and a moderate uptake in the liver (∼5 % IA g^−1^). Uptake in the wild‐type BON tumors may be explained by the expression of other relevant SSTR subtypes, such as SSTR3 and SSTR5.^[22]^ It is known from the literature that somatostatin analogs display the second highest affinity for SSTR5 and a moderate affinity for SSTR3.^[21a]^ These receptor subtypes are expressed at a comparatively high level in the wild‐type BON cell line, which most likely contributes to the uptake of our probe in the BON tumor. According to the PET/MRI and biodistribution data, the clearance route is predominantly renal with a high uptake in the kidneys (∼30 % IA g^−1^). Attaching a fluorophore to ^68^Ga‐DOTATATE usually causes higher renal uptake compared to the dye‐free ^68^Ga‐DOTATATE,[^21^, ^23^] leading to values of up to 70 % IA g^−1^.[^14b^, ^18a^] The same applies, usually to a smaller extent, for the uptake in the liver, which is also influenced by nonpolar structural moieties as typically given by fluorophores. In our study, the predominance of the renal clearance route compared to the hepatobiliary route may be attributed to the relatively hydrophilic indocarbocyanine dye with a short polymethine chain length, no additional benzene moieties and the presence of one sulfonate group. The tumor‐to‐background ratios 1 h after injection were moderate for the muscles and low for blood. Other studies with SSTR2‐targeting hybrid probes observed similar values 3–4 h after injection,[^14b^, ^18a^] which could be improved when imaging at later time points, such as 24 or 48 h after injection with ^67^Ga.^[18c]^

### Histological examination

**Figure 3:**
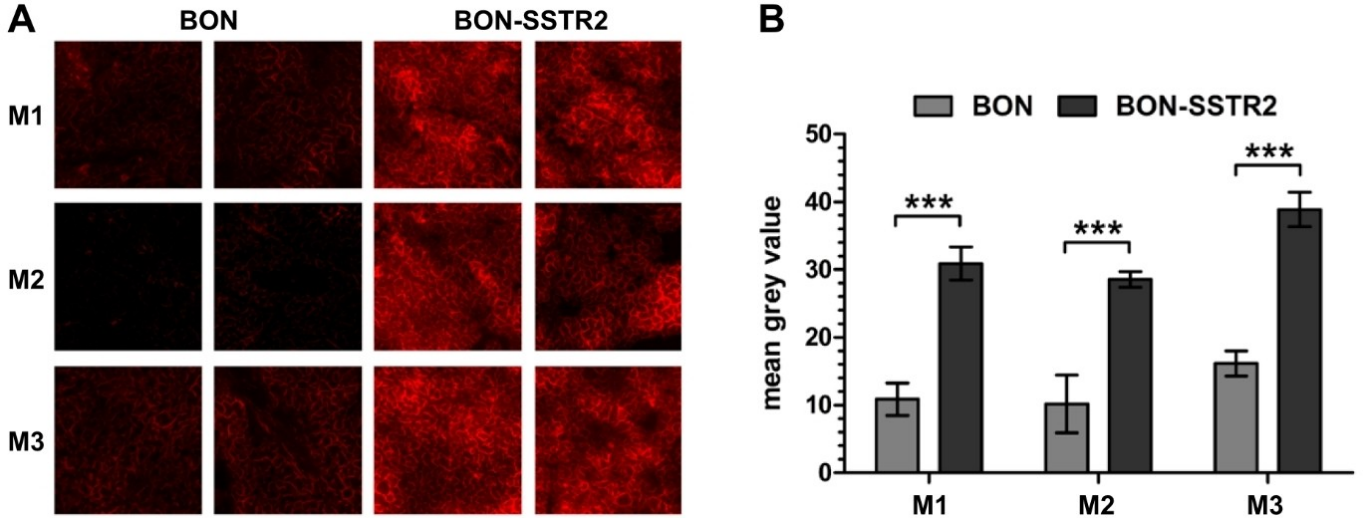
Histological *ex vivo* examination of BON wild‐type and SSTR2‐overexpressing BON tumors from xenograft‐bearing nude mice (*n*=3) injected with ^nat^Ga‐DOTA‐ICC‐TATE. A) Images from the confocal laser scanning microscopy. B) Quantitative analysis of the images. *** *P*=0.005.

When tumor sections of mice sacrificed 1 h p.i. were examined using a confocal laser microscope, no fluorescence above background level was detected. In particular, no differences between sections from SSTR2‐overexpressing and wild‐type tumor were found. We assumed that the tracer dose chosen for the PET/MRI scan was not high enough for fluorescence imaging on the histological level due to the inferior sensitivity of this method. We therefore repeated the experiment with the same tumor mouse model and injected 6 nmol of ^nat^Ga‐DOTA‐ICC‐TATE. Mice were sacrificed 5 h p.i., and the tumors excised. Upon confocal laser scanning microscopy of tumor sections from these mice (Figure 3A), we detected clear differences in the signals arising from the ICC dye in the probe between the SSTR2‐overexpressing (mean grey value 32.8±5.4) and the wild‐type BON tumor (mean grey value 12.4±3.3), found to be statistically significant (*P*=0.005). The necessity of using a higher dose for fluorescence microscopy due to its sensitivity is not unexpected as other studies have also used picomolar doses for PET or SPECT and nanomolar doses for fluorescence imaging or microscopy.[^7^, ^18b^, ^24^] Further studies are required to determine the optimal dose that can be used both for PET and for *ex vivo* examinations afterwards, e. g. for cancer staging or intraoperative frozen section analysis. However, increasing the amount of peptide for fluorescence imaging might weaken the image contrast for PET or SPECT due to a lower specific activity of the tracer.[^18b^, ^25^]

## Conclusion

We present a new modular design approach for the synthesis of multimodal imaging probes based on a bifunctional cyanine dye serving as a structural and functional linking unit. Our study demonstrates that a cyanine dye can serve as the linker in the comparatively compact SSTR2‐targeting hybrid probe ^68^Ga‐DOTA‐ICC‐TATE with direct proximity between dye and pharmacophore. Using radionuclide and fluorescence imaging modalities, we performed SSTR2‐imaging *in vitro, in vivo* and *ex vivo* in SSTR2‐expressing cell and tumor mouse models. Our dye‐bridged hybrid probe showed excellent *in vitro* SSTR2‐affinity and peptide functionality, whereas the *in vivo* PET imaging using ^68^Ga at an early imaging time point needs further improvements for higher tumor‐to‐background ratios. Histological *ex vivo* examination using fluorescence microscopy showed high specificity of the hybrid probe for the SSTR2‐overexpressing tumor in comparison to the wild‐type tumor.

While previous reports mainly focused on the use of Cy5‐ or Cy7‐based dyes for image‐guided surgery, we employed a Cy3‐based fluorophore due to chemical considerations during the establishment of the synthesis. In comparison to Cy7‐based probes, a Cy3‐based probe has its own eligibility because *in vitro* and histological *ex vivo* examinations after PET or SPECT studies can be easily performed using conventional fluorescence microscopes for detection. Future studies aim to expand our synthetic approach to a Cy5‐based construct with additional benefits for the field of image‐guided surgery.

## Experimental Section

### General information

Chemicals were purchased from commercial sources (like Sigma‐Aldrich, TCI, AlfaAesar, ABCR and Fluka). Indolenine precursors and the protected DOTA chelator were synthesized according to published procedures. ^1^H, ^13^C and ^71^Ga NMR spectra in solution were measured with the spectrometers ECX 400 (400 MHz) and ECP 500 (500 MHz) from JEOL and Avance 500 (500 MHz) and Avance 700 (700 MHz) from Bruker. Mass spectra were measured on a 6210 ESI‐TOF and 6230 ESI‐TOF from Agilent. UV/VIS spectra were recorded using a PerkinElmer LAMBDA 950 UV/Vis/NIR spectrometer and fluorescence spectra recorded using a JASCO FP‐6500 spectrometer. NP automated column chromatography was done on a CombiFlash Rf (Teledyne ISCO) using prepacked silica columns (30 μm). Purification of compounds using size exclusion chromatography was done on a Sephadex column (NAP‐25, Sephadex G‐25 DNA) with water as eluent.

### Synthesis

N*‐(Hydroxymethyl)‐phthalimide*: To a solution of phthalimide (10.0 g, 68.0 mmol, 1.0 equiv) in H_2_O (35 mL) was added a formaldehyde solution (37 % in H_2_O, 5.1 mL). After heating the suspension to 105 °C for 1 h under heavy stirring, a clear and colorless solution was obtained. Storing the solution in the freezer overnight led to the precipitation of a colorless solid, which was filtered off and washed with ice‐cold water. The filter cake was dried in vacuo at 30 °C and afforded the product as a colorless solid (10.9 g, 61.5 mmol, 90 %). ^1^H NMR ([D_6_]DMSO, 400 MHz): *δ*=7.93–7.88 (m, 4H; −CH), 6.39 (t, *J*=7.0 Hz, 1H; −OH), 4.96 (d, *J*=6.9 Hz, 2H; −CH_2_); ^13^C NMR ([D_6_]DMSO, 101 MHz): *δ*=167.4, 134.8, 131.6, 123.4, 60.1; MS (ESI+): *m*/*z* C_9_H_7_NO_3_ [*M*+Na]^+^ calculated: 200.0324, found: 200.0318.

*1,3,3‐Trimethyl‐2‐methylene‐5‐(phthal‐imidemethyl)‐indolenine*: Fischer's base (10.9 mL, 10.7 g, 51.6 mmol, 1.0 equiv) was dissolved in concentrated H_2_SO_4_ (55 mL). To the red‐brown solution, *N*‐(hydroxymethyl)phthalimide (10.9 g, 61.5 mmol, 1 equiv) was added portion‐wise over 45 min. The brown solution was stirred at room temperature for 75 h and poured onto ice‐cooled water (60 mL). After addition of ammonia solution (12.5 % in water, 400 mL) and filtration, the filter cake was dissolved in CH_2_Cl_2_ (350 mL) and washed with demineralized water (2×300 mL). The aqueous phase was extracted with dichloromethane (2×400 mL), dried over Na_2_SO_4_, filtered and the solvent removed under reduced pressure. After automated column chromatography (silica gel, eluent CH_2_Cl_2_/MeOH with 0–5 % and 0–0.5 % methanol), recrystallization with CH_2_Cl_2_/EtOH (1 : 1) and washing with methanol was done and afforded the product as a yellow to red solid with minor impurities (8.9 g, ca. 27 mmol, ca. 52 %). ^1^H NMR (CDCl_3_, 400 MHz): *δ*=7.81 (dd, *J*=5.4 Hz, *J*=3.0 Hz, 2H; −CH), 7.67 (dd, *J*=5.5 Hz, *J*=3.1 Hz, 2H; −CH), 7.24 (d, *J*=1.8 Hz, 1H; −CH), 7.18 (d, *J*=1.8 Hz, 1H; −CH), 6.44 (d, *J*=8.0 Hz, 1H; −CH), 4.74 (s, 2H; −CH_2_), 3.81 (s, 2H; −CH_2_), 2.98 (s, 3H; −CH_3_), 1.30 (s, 3H; −CH_3_); ^13^C NMR (CDCl_3_, 126 MHz): *δ*=168.3, 163.0, 146.3, 138.1, 134.0, 132.4, 128.9, 126.6, 123.4, 123.0, 104.7, 73.5, 44.2, 41.8, 30.0, 28.9; MS (ESI+): *m*/*z* C_21_H_20_N_2_O_2_ [*M*+H]^+^ calculated: 333.1604, found: 333.1649, [*M*+Na]^+^ calculated: 355.1423, found: 355.1468.

*Dye **1***: 5‐Carboxy‐1,3,3‐trimethyl‐2‐methylene‐1‐(4‐sulfobutyl)indolenine (1.12 g, 4.28 mmol, 1.0 equiv) and *N,N*‐diphenylformamidine (420 mg, 4.28 mmol, 1.0 equiv) were dissolved in acetic anhydride (34.3 mL) and heated to 120 °C for 20 min. To the red‐brown solution was then added a suspension of 1,3,3‐trimethyl‐2‐methylene‐5‐(phthalimidomethyl)indoline (1.02 g, 4.28 mmol, 1.0 equiv) and sodium acetate (525 mg, 12.8 mmol, 3.0 equiv) in acetic anhydride (17.1 mL). Afterwards, acetic acid (1.55 mL) was added dropwise, which led to a color change to pink‐violet. The reaction mixture was stirred at 120 °C for 30 min. The solvent was removed under reduced pressure and the residue taken up in DMF for precipitation of the dye by adding diethyl ether (3x). The precipitate was purified by automated column chromatography (silica gel, eluent dichloro‐methane/methanol with 0–50 % methanol) and afforded product 1 as a pink‐red solid (1.01 g, 1.48 mmol, 35 %). *R*_f_=0.5 (CH_2_Cl_2_/MeOH 1 : 1); ^1^H NMR (CD_3_OD, 500 MHz): *δ*=8.57 (t, *J*=13.5 Hz, 1H; −CH), 8.16–8.12 (m, 2H; −CH), 7.94–7.89 (m, 2H; −CH), 7.89–7.83 (m, 2H; −CH), 7.64 (d, *J*=1.7 Hz, 1H; −CH), 7.53 (dd, *J*=8.2 Hz, *J*=1.7 Hz, 1H; −CH), 7.44 (d, *J*=8.7 Hz, 1H; −CH), 7.40 (d, *J*=8.3 Hz, 1H; −CH), 6.59 (d, *J*=13.6 Hz, 1H; −CH), 6.55 (d, *J*=13.3 Hz, 1H; −CH), 4.95 (s, 2H; −CH_2_), 4.21 (t, *J*=7.4 Hz, 2H; −CH_2_), 3.75 (s, 3H; −CH_3_), 2.95 (t, *J*=6.8 Hz, 2H; −CH_2_), 2.08–1.98 (m, 2H; −CH_2_), 1.82 (s, 6H; −CH_3_), 1.79 (s, 6H; −CH_3_); ^13^C NMR ([D_6_]DMSO, 176 MHz): *δ*=175.5, 173.3, 167.7, 167.0, 149.8, 145.4, 141.9, 141.2, 140.4, 134.6, 131.6, 130.6, 127.8, 123.3, 121.7, 112.1, 110.9, 104.7, 102.7, 50.8, 49.2, 48.3, 43.7, 40.8, 31.8, 27.5, 27.1, 26.0, 22.5; MS (ESI+): *m*/*z* C_38_H_39_N_3_O_7_S [*M*+H]^+^ calculated: 682.2588, found: 682.2581, [*M*+Na]^+^ calculated: 704.2406, found: 704.2408.

*Dye **2***: Dye **1** (50.0 mg, 73.3 μmol, 1.0 equiv) was dissolved in methanol (3.75 mL, purged with Argon) and hydrazine monohydrate (10.7 μL, 11.0 mg, 220 μmol, 6.0 equiv) was added in two portions over 15 min. The reaction mixture was heated to 90 °C in the microwave for 30 min. The solvent was removed under reduced pressure. Purification of the residue by column chromatography (silica gel, eluent CH_2_Cl_2_/MeOH with 0–60 % methanol) afforded product **2** as a pink‐red solid (32.5 mg, 58.9 μmol, 80 %). *R*_f_=0.05 (CH_2_Cl_2_/MeOH 1 : 1); ^1^H NMR (CD_3_OD, 500 MHz): *δ*=8.57 (t, *J*=13.5 Hz, 1H; −CH), 8.11–8.07 (m, 2H; −CH), 7.60 (d, *J*=1.7 Hz, 1H; −CH), 7.47 (d, *J*=7.9 Hz, 1H; −CH), 7.38 (d, *J*=3.6 Hz, 1H; −CH), 7.36 (d, *J*=3.1 Hz, 1H; −CH), 6.55 (d, *J*=10.3 Hz, 1H; −CH), 6.52 (d, *J*=10.6 Hz, 1H; −CH), 4.21 (t, *J*=7.3 Hz, 2H; −CH_2_), 3.95 (s, 2H; −CH_2_), 3.73 (s, 3H; −CH_3_), 2.96 (t, *J*=6.7 Hz, 2H; −CH_2_), 2.07–2.01 (m, 2H; −CH_2_), 1.82 (s, 6H; −CH_3_), 1.81 (s, 6H; −CH_3_); ^13^C NMR (CD_3_OD, 176 MHz): *δ*=176.9, 176.3, 152.2, 144.9, 143.5, 142.6, 141.5, 139.3, 136.7, 131.7, 129.7, 124.4, 123.2, 112.4, 111.4, 104.6, 104.3, 51.6, 50.7, 50.3, 45.8, 45.1, 31.9, 30.8, 28.4, 28.2, 27.1, 23.5; UV/Vis (H_2_O): *λ*_max_ (*ϵ*)=551 nm (130 000 L mol^−1^ cm^−1^); fluorescence (H_2_O): *λ*_ex_=530 nm, *λ*_em_=570 nm; MS (ESI+): *m*/*z* C_30_H_37_N_3_O_5_S [*M*+H]^+^ calculated: 552.2454, found: 552.2515, [*M*+Na]^+^ calculated: 574.2346, found: 574.2332.

*DOTA‐ICC label **3***: The *tert*‐butyl protected DOTA chelator (68.5 mg, 109 μmol, 1.2 equiv) and HSTU (45.5 mg, 127 μmol, 1.4 equiv) were dissolved in DMF (750 μL) and DIPEA (23.1 μL, 17.6 mg, 136 μmol, 1.5 equiv) was added. The reaction mixture was stirred for 1 h at room temperature. Compound **2** (50.0 mg, 90.6 μmol, 1.0 equiv) was dissolved in demineralized water (1.2 mL) and added with DIPEA (46.2 μL, 35.1 mg, 272 μmol, 3.0 equiv) to the reaction mixture. After stirring for 15 h at room temperature, the solvent was removed under reduced pressure. The residue was purified twice by automated column chromatography (silica gel, eluent CH_2_Cl_2_/MeOH with 0–100 % methanol) and afforded product **3** as a pink‐red solid (59.5 mg, 51.1 μmol, 56 %). *R*_f_=0.3 (CH_2_Cl_2_/MeOH 1 : 1); ^1^H NMR (CD_3_OD, 400 MHz): *δ*=8.55 (t, *J*=13.4 Hz, 1H; −CH), 8.13–8.09 (m, 2H; −CH), 7.56 (d, *J*=1.5 Hz, 1H; −CH), 7.44–7.37 (m, 3H; −CH), 6.61 (d, *J*=13.7 Hz, 1H; −CH), 6.55 (d, *J*=13.2 Hz, 1H; −CH), 4.50 (b, 2H; −CH_2_), 4.19 (t, *J*=6.7 Hz, 2H; −CH_2_), 3.75 (s, 3H; −CH_3_), 3.64–3.37 (m, 4H; −CH_2_), 3.21–3.00 (b, 4H), 2.95–2.89 (m, 4H; −CH_2_), 2.83–2.37 (m, 8H; −CH_2_), 2.36–2.14 (b, 4H; −CH_2_), 2.07–1.92 (m, 6H; −CH_2_), 1.79 (s, 6H; −CH_3_), 1.78 (s, 6H; −CH_3_), 1.58–1.55 (m, 2H; −CH_2_), 1.50 (s, 9H; −CH_3_), 1.48 (s, 18H;‐ CH_3_); ^13^C NMR (CD_3_OD, 176 MHz): *δ*=177.8, 175.5, 174.5, 174.4, 174.2, 171.8, 152.4, 147.2, 143.1, 142.8, 141.9, 138.7, 132.4, 129.3, 124.6, 123.0, 112.8, 111.8, 105.8, 104.2, 82.8, 57.3, 56.8, 51.6, 51.0, 50.0, 49.9, 45.0, 43.9, 43.4, 32.3, 28.5, 28.1, 27.0, 23.5; MS (ESI+): C_60_H_90_N_8_O_13_S [*M*+Na]^+^ calculated: 1185.6246, found: 1185.6234, [*M*+2Na‐3 C_4_H_8_]^2+^ calculated: 520.2127, found: 520.2144.

^*nat*^ *Ga‐DOTA‐ICC label*: A mixture of TFA/demineralized water/phenol/ thioanisol/1,2‐ethandithiol (82.5 : 5 : 5 : 5 : 2.5, 1.5 mL) was added to the DOTA‐ICC label **3** (61.6 mg, 52.9 μmol, 1.0 equiv) and stirred for 4 h at room temperature. The solvent was removed under reduced pressure, the residue taken up with water and washed with CH_2_Cl_2_. The aqueous phase was lyophilized. Preparative HPLC was performed in an isocratic mode on a high‐pressure gradient system (stainless steel), equipped with a Shimadzu LC‐8 A pump, Shimadzu CBM‐20 A controller, variable wavelength UV detector from Knauer and a Rheodyne injector with 10 mL sample loop. The stationary phase was a prepacked LUNA HILIC diol column (200 Å, 5 μM, 250×21.2 mm) from Phenomenex with precolumn. The eluent was a degassed MeCN/water mixture (75 % MeCN+50 mM ammonium formate) and a flow rate of 20 mL min^−1^ was applied. Detection was performed at 480 nm. The appropriate fractions were lyophilized to afford the deprotected DOTA‐ICC label (14.9 mg), which was then dissolved in water (800 μL). GaCl_3_ was added and the pH value adjusted to 4 using NaOH. The reaction mixture was heated at 80 °C for 30 minutes. The mixture was diluted with water, dialyzed (regenerated cellulose (Carl Roth), MWCO=500 g mol^−1^, water, 3 d) and lyophilized to afford the product as a pink‐red solid (14.0 mg, 13.2 μmol, 25 % over 2 steps). ^1^H NMR (D_2_O/CD_3_CN 5 : 2, 700 MHz): *δ*=8.80 (t, *J*=13.5 Hz, 1H), 8.35 (d, *J*=1.6 Hz, 1H), 8.35–8.33 (m, 1H), 7.77 (d, *J*=1.7 Hz, 1H), 7.68 (dd, *J*=8.1, 1.6 Hz, 1H), 7.65 (dd, *J*=11.9, 8.2 Hz, 2H), 6.73 (d, *J*=13.7 Hz, 1H), 6.66 (d, *J*=13.2 Hz, 1H), 4.76 (d, *J*=3.3 Hz, 2H), 4.40 (t, *J*=7.4 Hz, 2H), 4.27 (dd, *J*=15.2, 3.8 Hz, 3H), 4.24 (s, 2H), 4.15 (d, *J*=11.2 Hz, 2H), 4.12 (s, 1H), 4.10 (s, 1H), 4.06 (s, 3H), 4.03 (s, 3H), 3.95 (s, 3H), 3.76 (s, 3H), 3.74–3.72 (m, 3H), 3.59 (ddt, *J*=14.3, 8.3, 4.4 Hz, 6H), 3.50 (td, *J*=14.3, 4.4 Hz, 2H), 3.22 (t, *J*=7.4 Hz, 2H), 2.21 (dq, *J*=21.9, 7.6 Hz, 4H), 2.06 (s, 6H), 2.04 (s, 6H); ^13^C NMR (D_2_O/d_3_‐MeCN 5 : 2, 176 MHz): *δ*=176.6, 174.6, 173.5, 173.2, 171.5, 171.0, 169.1, 151.1, 145.5, 142.1, 141.8, 140.9, 136.6, 131.1, 130.0, 128.1, 123.6, 121.6, 111.9, 110.8, 104.1, 102.5, 63.4, 61.5, 59.7, 57.4, 57.3, 54.7, 54.5, 50.7, 49.8, 49.0, 44.0, 42.8, 42.5, 39.2, 31.7, 27.6, 27.2, 26.0, 22.1; ^71^Ga NMR (D_2_O, 153 MHz): *δ*=‐25.0; UV/Vis (H_2_O): *λ*_max_ (*ϵ*)=552 nm (80 000 L mol^−1^ cm^−1^); fluorescence (H_2_O): *λ*_ex_=530 nm, *λ*_em_=570 nm; MS (ESI+): C_48_H_63_GaN_8_O_13_S [*M*+Na]^+^ calculated: 1082.3305, found: 1082.3322; MS (ESI‐): C_48_H_63_GaN_8_O_13_S; [*M*‐H]^+^ calculated: 1059.3413, found: 1059.3402.

*DOTA‐ICC‐TATE conjugate*: The peptide conjugate was synthesized in a 0.05 mmol scale on a Thr‐preloaded Wang Resin. The synthesis was carried out on a PTI synthesizer (Protein Technologies, USA) with double couplings of each amino acid (5 equiv. amino acid for 40 min) in DMF. Both cysteines were introduced as MMT protected building blocks. The DOTA‐ICC label **3** (100 μmol, 2.0 equiv) was coupled manually using HATU (100 μmol, 2.0 equiv) and DIPEA (200 μmol, 4.0 equiv) in DMF (2 mL) for 4 h. Afterwards, MMT protecting groups were cleaved by treating the resin 5x with a mixture of CH_2_Cl_2_/TFA/TIS (94 : 1 : 5; 3 mL) for 2 min. Followed by a CH_2_Cl_2_ and DMF wash, the cyclization using *N‐*chlorosuccinimide (2 equiv) in DMF for 15 min was carried out. The final cleavage from the resin was done in TFA/TIS/H_2_O (95 : 2.5 : 2.5) for 3 h. The crude DOTA‐ICC‐TATE conjugate was purified by preparative HPLC (RP‐C18, 0–5 min 95 : 5, water (0.1 % TFA)/MeCN (0.1 % TFA); 5–60 min 10 : 90, water (0.1 %TFA)/MeCN (0.1 % TFA)) using a Gilson PLC 2020 personal Purification System (Gilson Inc., Middleton, WI, USA) including a Nucleodur column (VP250/32 C18 HTec, 5 μm) from Macherey‐Nagel with a flow rate of 30 mL min^−1^. The product was gained in two peaks as a pink powder (9.5 mg, 4.7 μmol, 5 %) and analyzed by UPLC‐UV using an Aquity UPLC H‐Class with a quaternary solvent manager, a Waters autosampler and an Aquity UPLC‐BEH RP‐C18 column (1.7 μm, 2.1×50 mm) from Waters with a flow rate of 0.6 mL min^−1^ connected to a Waters UV detector and a QDa detector and the following gradient used with solvents A and B (A=H_2_O+0.1 %TFA; B=MeCN+0.1 %TFA): 0–1.5 min (with 5 % B in A); from 1.5–13 min (5 % B to 95 % B in A); 13–13.9 min (with 95 % B in A); 13.91–15 min (with 5 % B in A). Detection was performed at 220 nm. UV/Vis (H_2_O): *λ*_max_ (*ϵ*)=553 nm (74 000 L mol^−1^ cm^−1^); fluorescence (H_2_O): *λ*_ex_=530 nm, *λ*_em_=570 nm; *m*/*z* C_97_H_128_N_18_O_24_S_3_ [*M*+2H]^2+^ calculated: 1013.93, found: 1013.81 and 1013.62, [*M*+H+Na]^2+^ calculated: 1024.93, found: 1024.75 and 1025.01, [*M*+3H]^3+^ calculated: 676.29, found: 676.25 and 676.35.

^*nat*^ *Ga‐DOTA‐ICC‐TATE conjugate*: GaCl3 (217 μg, 1.24 nmol, 5.0 equiv) in 205.6 μL HEPES buffer and 25 μL MeCN was added to DOTA‐ICC‐TATE (500 μg, 247 nmol, 1.0 equiv). The pH of the solution was adjusted to pH 4 using HCl (1 M) and the reaction mixture heated to 90 °C for 1 h. After cooling down, the purification was done by using a Sephadex column and water as eluent. The fractions were lyophilized, dissolved in 1.0 mL water and the concentration was determined via absorption spectroscopy using an absorption coefficient of 80 000 L mol^−1^ cm^−1^ to be 64 nmol. Analytical RP‐HPLC of the ^nat^Ga‐labeled peptide conjugate in comparison to the Ga‐free peptide conjugate was performed on an Agilent 1200 system (Agilent, Waldbronn, Germany) equipped with an Eclipse XDB−C18 bonded silica (5 μm, 50×4.6 mm) column (Agilent, Waldbronn, Germany) with a flow rate of 1 mL min^−1^ and the column at 55 °C. Elution was performed using a linear gradient with solvents A and B (A=H_2_O+0.1 %TFA; B=MeCN+0.1 %TFA) with a gradient of 20–60 % B in A over 20 min. Detection was performed at 543 nm.

### Radiolabeling with ^68^Ga

Radiolabeling experiments were performed on a synthesis module (Modular Lab PharmTracer) which allows fully automated cassette‐based labelling of Gallium tracers utilizing a Pharmaceutical grade ^68^Ge/^68^Ga Generator (GalliaPharm, 1.85 GBq, GMP) both purchased from Eckert & Ziegler GmbH. Cassettes were GMP‐Conform, sterile and used without pre‐conditioning of the cartridges. Gallium‐generator at 1‐month post calibration was eluted with aqueous HCl (0.1 M, 7 mL) and the eluate was purified on an ion‐exchange cartridge followed by elution directly into the reactor pre‐heated at 40 °C using 1 mL of 0.1 M HCl in acetone. An aliquot of DOTA‐ICC‐TATE, 20 μg (stock solution, 1 μg/μL in water) was mixed with 500 μL HEPES buffer (0.1 M in WFI, pH 7) and added to the reactor before starting the synthesis. The reaction mixture (pH 3–4) was then heated for 500 s at 95 °C. After the reaction, the reactor was cooled with 0.5 mL of saline following the transfer of the contents of the reactor on a C_18_ cartridge (SEPPAK, Waters GmbH) for post‐purification. After washing the C_18_ cartridge with saline, the product was eluted with 1 mL of ethanol/water (1 : 1) and diluted with 1.5 mL saline for animal experiments. RP‐HPLC (Knauer) with a Eurospher II column (C_18_, 250×4 mm) was used to quantify the radiochemical purity of ^68^Ga‐DOTA‐ICC‐TATE. The HPLC was equipped with an Azura P.6.1 L pump coupled with ultraviolet (Azura UVD 2.1L) and radiometric (γ‐Raytest‐Isotopenmessgeräte GmbH) detectors. The gradient elution system used mobile phase A (100 % acetonitrile) and mobile phase B (deionized H_2_O containing 0.1 % trifluoroacetic acid) and a flow rate of 1.0 mL/min. Starting with 0 % A and 100 % B, the gradient was increased to 100 % A over 25 min and finally returned to initial gradient conditions over 5 minutes. Radiochemical purity of the tracer was found to be≥95 %, radiochemical yield was about 40 % and specific activity was calculated as 40 GBq/μmol of peptide.

### *In vitro* tests

*Toxicity tests*: BON‐SSTR2 cells were seeded in quadruplicates in 96 well plates at a density of 5,000 cells per well and grown overnight. Cells were treated with the indicated concentrations of DOTA‐ICC‐TATE in 100 μL medium per well. Metabolic activity and cell number were determined after another 96 h. For this, 100 μL medium containing AlamarBlue™ redox indicator (ThermoFisher) were added on top of each well, incubated for 3–4 h and the resulting fluorescence was measured using an EnVision Multilabel Plate Reader (PerkinElmer). Afterwards, the supernatant was removed; cells were fixated with 4 % *v*/*v* formaldehyde for 10 min and stained with 1 μg/mL DAPI in PBS/0.1 % *v*/*v* Triton for another 10 min. Four fields per well were imaged using an IN Cell Analyzer 1000 (GE Healthcare) with a 4x objective and nuclei were counted by Investigator software (GE Healthcare). All values were normalized to the control treated with vehicle and analyzed using GraphPad Prism 5.04.

*Endocytosis assay*: Rat insulinoma RIN1038 cells stably expressing a ratSSTR2‐GFP fusion protein almost exclusively show green cell membrane fluorescence in the resting state while in the presence of a functional agonist, the receptor‐GFP fusion protein is translocated to a perinuclear endocytic vesicle compartment. This shift is readily recognized by inspection of the fluorescent image and can be detected by software‐based quantitation algorithms. After an incubation time of 30 min with 1 μM DOTA‐ICC‐TATE in RPMI1640 medium at 37 °C, cells were fixed for 10 min using 4 % formaldehyde in PBS, air‐dried and mounted on glass slides with ImmuMount (ThermoFisher Scientific). Mounted cells were imaged using a confocal laser‐scanning microscope (LSM510, Carl Zeiss) with a helium–neon laser at 488 and 543 nm, BP505‐525 and LP560 emission filters and a 63x NeoFluar oil immersion objective. For quantitative analysis and determination of the concentration of half‐maximal internalization effect (EC_50_), eleven concentrations of DOTA‐ICC‐TATE (3 pM–300 nM) were used in the same cell model in quadruplicates in a 96 well plate. After an incubation time of 30 min with 1 μM DOTA‐ICC‐TATE in RPMI1640 medium at 37 °C, cells were fixed for 10 min using 4 % formaldehyde plus 0.1 μg mL^−1^ DAPI in PBS before adding 100 μL of PBS per well. For automated microscopy, microscopic fluorescence images were recorded by an IN Cell Analyzer 1000 (GE Healthcare) with 20x magnification at 5 frames per well in a 96 well format. Image processing was performed with In Cell Investigator software (GE Healthcare) applying the provided granularity algorithm. The output of the analysis was indicated as vesicle area/cell and was utilized for calculation of concentration‐response curves resulting in the determination of half‐maximal activity (EC_50_).

*In vitro binding affinity assay*: 10 nmol Tyr^11^‐somatostatin‐14 (Tyr^11^‐SST14, Bachem, Bubendorf, Switzerland) were iodinated by the chloramine T method^[26]^ with 1 mCi carrier‐free Na^125^I (NEZ033 L010MC, PerkinElmer) and purified by HPLC (Analytic HPLC 1200 Series, Agilen). For competitive radioligand binding assays, 40 000 BON cells or BON‐SSTR2 cells (overexpressing human SSTR2)^[22]^ were seeded in 96‐well plates and grown overnight. The next day, cells were incubated in binding buffer (50 mM HEPES, pH 7.4, 5 mM MgCl_2_, 1 mM CaCl_2_, 0.5 % *w*/*v* BSA, complete protease inhibitors (Roche)) containing 100 000 counts per minute (cpm) [^125^I]‐Tyr^11^‐SST14 and increasing concentrations of unlabeled peptide. After 30 min at 37 °C, cells were washed with ice‐cold washing buffer (50 mM Tris⋅HCl, pH 7.4, 125 mM NaCl, 0.05 % w/v BSA), lysed with 1 N NaOH, transferred to vials and measured in a gamma counter (Wallac 1470 Wizard, PerkinElmer). The obtained cpm values were analyzed with GraphPad Prism 5.04 and IC_50_ values were calculated by nonlinear regression (one site‐fit logIC_50_, least squares fit).

### *In vivo* imaging and biodistribution

*Tumor model*. Neuroendocrine BON and BON‐SSTR2 cells (3x10^6^) were suspended in a volume of 150 μL 0.9 % NaCl and inoculated subcutaneously on the right and left shoulder of female nude NMRI*‐Foxn1^nu^/Foxn1* ^*nu*^ mice (Janvier Labs, Saint‐Berthevin, France). After 2–3 weeks of tumor growth, the tumor size was sufficient for imaging. Animal care followed institutional guidelines, and all experiments were approved by local animal research authorities (approval no. G0192/08).

*PET/MRI imaging*. Tomographic imaging was performed using the dedicated small animal 1 Tesla nanoScan PET/MRI (Mediso, Hungary). Tumor‐bearing mice (*n*=3) were anaesthetized with isoflurane and were given an injection of 8.8–14.3 MBq ^68^Ga‐DOTA‐ICC‐TATE in a volume of 150 μL into the tail vein for PET. PET scans were performed for 20 min starting 41–48 min after injection of the probe. The uptake of ^68^Ga‐DOTA‐ICC‐TATE in the BON and BON‐SSTR2 tumors was determined by manual contouring of a volume‐of‐interest (VOI) of the PET image using PMOD 3.5 (PMOD Technologies Ltd., Switzerland). An average standard uptake value was computed from the 10 hottest voxels within the ^68^Ga‐DOTA‐ICC‐TATE positive lesions. MRI‐based attenuation correction of the PET was conducted with the T1‐weighted material map (matrix 144×144×164, with dimensions 0.5×0.5×0.6 mm^3^, TR:15 ms, TE 2.1 ms and a flip angle of 25°). Further anatomic MRI scans were acquired using a T2‐weighted 2D fast‐spin echo sequence (T2‐FSE 2D) with the following parameters: transverse sequentially, 224×224×26, 0.3×0.3×1.0 mm^3^, TR: 5409 ms, TE: 84 ms and a flip angle of 180°.

*Biodistribution study*: Tumor‐bearing mice (*n*=3) were injected with approximately 5 MBq of ^68^Ga‐DOTA‐ICC‐TATE in a volume of 150 μL 0.9 % NaCl into a lateral tail vein via a catheter. Mice were sacrificed by cervical dislocation and dissected 1 h after injection. Tumors, blood, stomach, pancreas, small intestine, colon, liver, spleen, kidney, heart, lung, muscle and femur samples were weighed and uptake of radioactivity was measured by a gamma counter (Wallac 1470 Wizard, PerkinElmer).

**Histological** ***ex vivo*** **examination**: Tumor‐bearing mice (*n*=3) were injected with approximately 6 nmol of ^nat^Ga‐DOTA‐ICC‐TATE in a volume of 150 μL 0.9 % NaCl into a lateral tail vein. Mice were sacrificed and dissected 5 h after injection. Sections of tumors were fixed for 10 min using 4 % formaldehyde in PBS, air‐dried and mounted on glass slides with ImmuMount (ThermoFisher Scientific). Tissues were imaged using a confocal laser‐scanning microscope (LSM510, Carl Zeiss) with a helium‐neon laser at 543 nm, LP560 emission filter and a 63x NeoFluar oil immersion objective.

## Conflict of interest

The authors declare no conflict of interest.

## Supporting information

As a service to our authors and readers, this journal provides supporting information supplied by the authors. Such materials are peer reviewed and may be re‐organized for online delivery, but are not copy‐edited or typeset. Technical support issues arising from supporting information (other than missing files) should be addressed to the authors.

Supplementary
