# Supplementary material for: A Cyanine‐Bridged Somatostatin Hybrid Probe for Multimodal SSTR2 Imaging in Vitro and in Vivo: Synthesis and Evaluation
Source: Chembiochem. 2021 Jan 4;22(7):1307–15. doi: 10.1002/cbic.202000791 (PMC8048842; doi:10.1002/cbic.202000791)

ChemBioChem

Supporting Information

A Cyanine-Bridged Somatostatin Hybrid Probe for Multimodal SSTR2 Imaging in Vitro and in Vivo: Synthesis and Evaluation

Isabelle Heing-Becker, Carsten Grötzinger, Nicola Beindorff, Sonal Prasad, Sarah Erdmann, Samantha Exner, Rainer Haag, and Kai Licha*

Author Contributions

I.H.-B. Conceptualization:Lead; Data curation:Equal; Formal analysis:Equal; Investigation:Lead; Methodology:Equal; Project administration:Equal; Validation:Equal; Visualization:Lead; Writing – original draft:Lead
C.G. Data curation:Equal; Formal analysis:Equal; Investigation:Equal; Methodology:Equal; Resources:Equal; Validation:Equal; Visualization:Equal; Writing – original draft:Supporting; Writing – review & editing:Lead
N.B. Data curation:Equal; Formal analysis:Supporting; Investigation:Equal; Resources:Lead; Visualization:Supporting; Writing – review & editing:Supporting
S.P. Data curation:Supporting; Formal analysis:Supporting; Investigation:Equal; Resources:Equal; Writing – review & editing:Supporting
S.E. Data curation:Supporting; Investigation:Supporting; Writing – review & editing:Supporting
S.E. Data curation:Supporting; Investigation:Supporting; Writing – review & editing:Supporting
R.H. Funding acquisition:Lead; Project administration:Equal; Resources:Equal; Supervision:Lead; Writing – review & editing:Equal
K.L. Conceptualization:Lead; Formal analysis:Equal; Methodology:Equal; Project administration:Equal; Resources:Equal; Supervision:Lead; Writing – review & editing:Lead

Table of Contents

Abbreviations

1. Spectroscopic characterization of labels and conjugates
2. *In vitro* tests
3. PET/MRI scans
4. ^{1}H and ^{13}C NMR spectra
5. Mass spectra
6. Chromatograms

Abbreviations

DCM	dichloromethane
DIPEA	*N,N*-diisopropylethylamine
DMF	*N,N*-dimethylformamide
DMSO	dimethyl sulfoxide
DOTA	1,4,7,10-tetraazacyclododecane-1,4,7,10-tetraacetic acid
eq.	equivalent(s)
ESI	electrospray ionization
HEPES	2-[4-(2-hydroxyethyl)piperazin-1-yl]ethanesulfonic acid
HPLC	high performance liquid chromatography
HSTU	*N,N,N',N'*-tetramethyl-O-(N-succinimidyl)uroniumhexafluorophosphate
ICC	indocarbocyanine
MMT	4-methoxy trityl
MS	mass spectrometry
MWCO	molecular weight cut-off
NMR	nuclear magnetic resonance
NP	normal phase
PBS	phosphate buffered saline
RP	reverse phase
TFA	trifluoroacetic acid
TIS	triisopropylsilane

1. Spectroscopic characterization of labels and conjugates

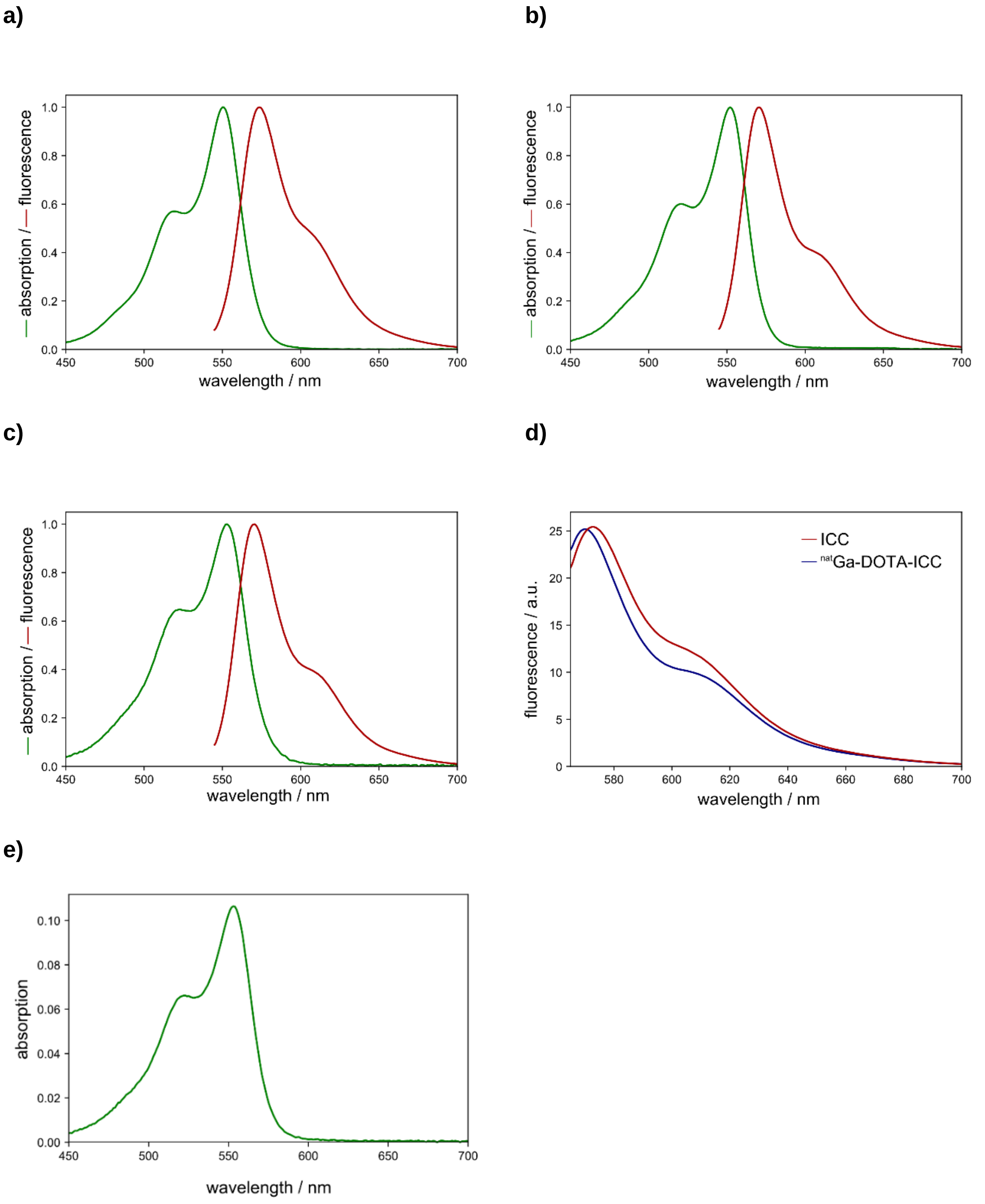

Figure S1. UV/VIS absorption and fluorescence spectra (λ_{ex}= 530 nm) for a) – d) were measured in water for concentrations between 1 – 5 µM for the absorption spectra and with 5 µM for fluorescence spectra. a) Normalized spectra for ICC dye **2** with $\lambda_{abs,max}$ = 551 nm, $\lambda_{em,max}$ = 570 nm and ε = 130.000 L/(mol cm). b) Normalized spectra for ^{nat}Ga-DOTA-ICC with $\lambda_{abs,max}$ = 552 nm, $\lambda_{em,max}$ = 570 nm and ε = 80.000 L/(mol cm). c) Normalized spectra for DOTA-ICC-TATE conjugate with $\lambda_{abs,max}$ = 553 nm, $\lambda_{em,max}$ = 570 nm and ε = 74.000 L/(mol cm). d) Comparison of the fluorescence spectra of 5 µM solutions (in water) of the ICC dye **2** and the ^{nat}Ga-DOTA-ICC label, where fluorescence behaviour is not significantly changed in the ^{nat}Ga-containing multimodal label. e) UV/VIS absorption spectrum of the ^{nat}Ga-DOTA-ICC-TATE conjugate in water.

2. *In vitro* tests

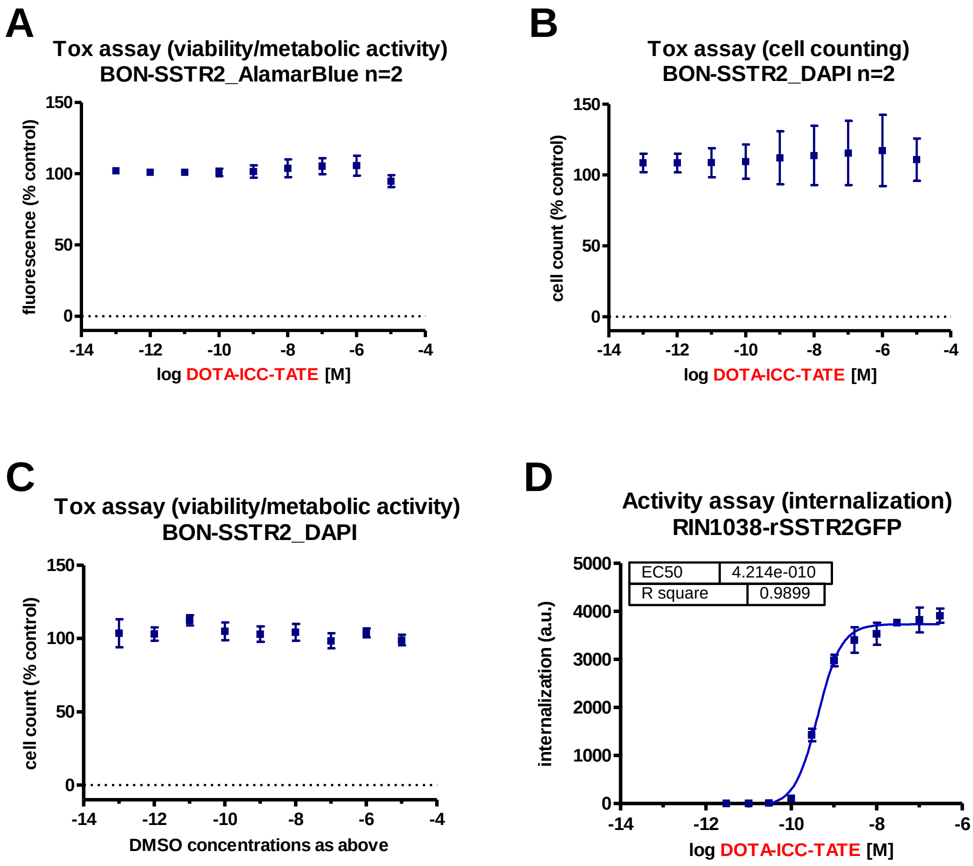

Figure S2. Toxicity assays (A-C) and endocytosis assay (D) of the DOTA-ICC-TATE conjugate.

3. PET/MRI scans

Mouse 1:

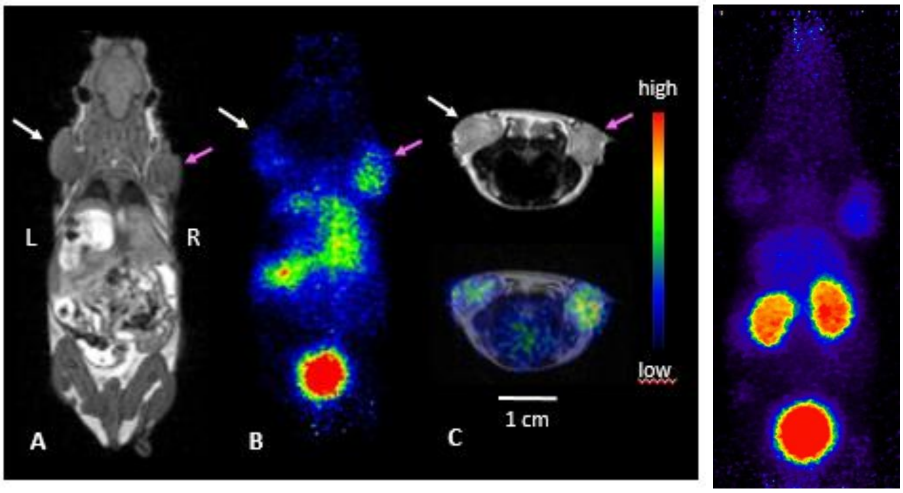

Figure S3. PET/MRI images of BON tumors (white arrows) and BON-SSTR2 tumors (pink arrows) and corresponding MIP 41 min after intravenous injection of 8.8 MBq ^{68}Ga-DOTA-ICC-TATE. The scale bar is representative for all images.

A: T1 coronal MRI; B: ^{68}Ga-DOTA-ICC-TATE PET coronal image; C: T2 FSE 2D transverse MRI, and fused with PET image.

BON tumor: Uptake = 4.95% IA/mL SSTR2 tumor: Uptake = 7.93% IA/mL

Mouse 2:

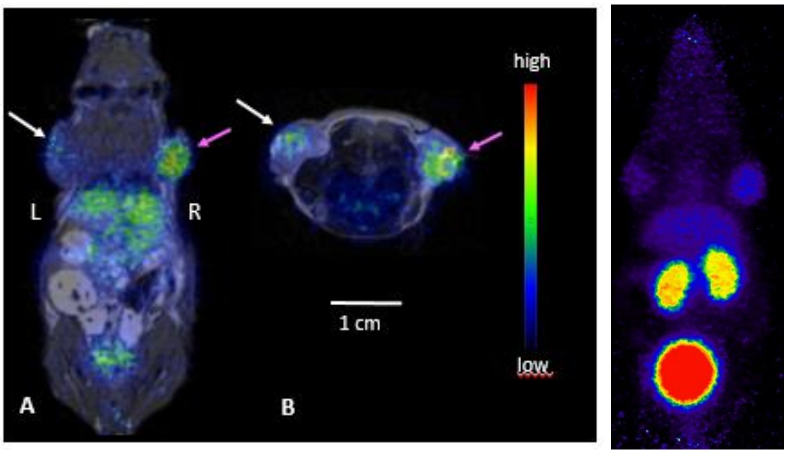

Figure S4. PET/MRI images of BON tumors (white arrows) and BON-SSTR2 tumors (pink arrows) and corresponding MIP 43 min after intravenous injection of 9.1 MBq ^{68}Ga-DOTA-ICC-TATE. The scale bar is representative for all images.

A: T1 coronal MRI fused with ^{68}Ga-DOTA-ICC-TATE PET image; B: T2 FSE 2D transverse MRI fused with PET image.

BON tumor: Uptake = 4.75% IA/mL SSTR2 tumor: Uptake = 7.15% IA/mL

Mouse 3:

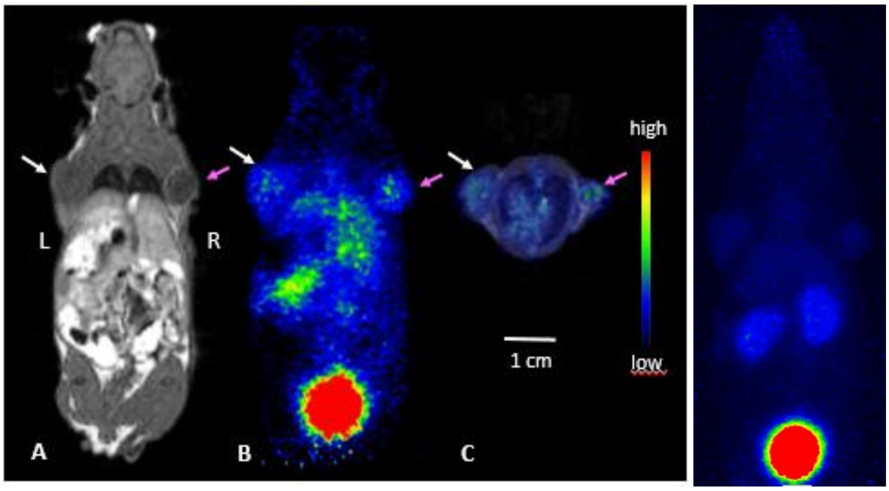

Figure S5. PET/MRI images of BON tumors (white arrows) and BON-SSTR2 tumors (pink arrows) and corresponding MIP 48 min after intravenous injection of 14.3 MBq ^{68}Ga-DOTA-ICC-TATE. The scale bar is representative for all images.

A: T1 coronal MRI; B: ^{68}Ga-DOTA-ICC-TATE PET coronal image; C: T1 transverse MRI fused with PET image.

BON tumor: Uptake = 4.15% IA/mL SSTR2 tumor: Uptake = 4.82% IA/mL

4. ^{1}H and ^{13}C NMR spectra

NMR spectra of *N*-(Hydroxymethyl)-phthalimide

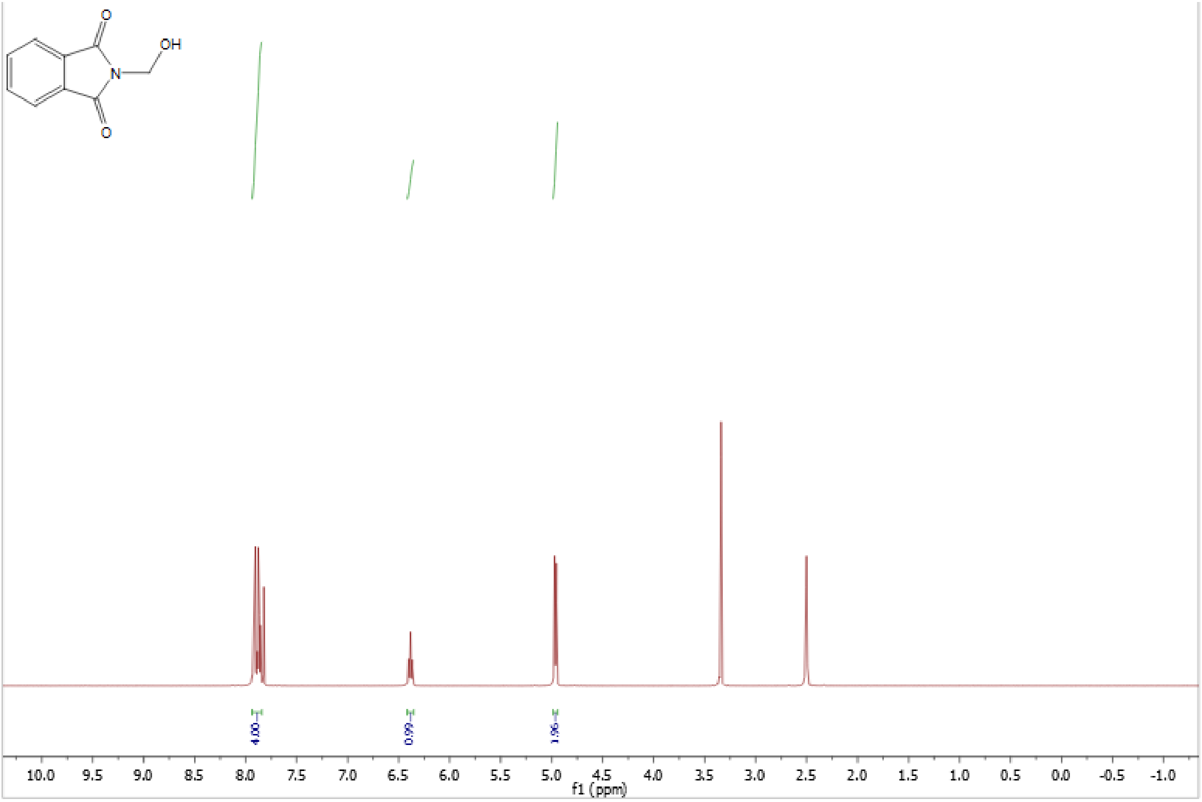

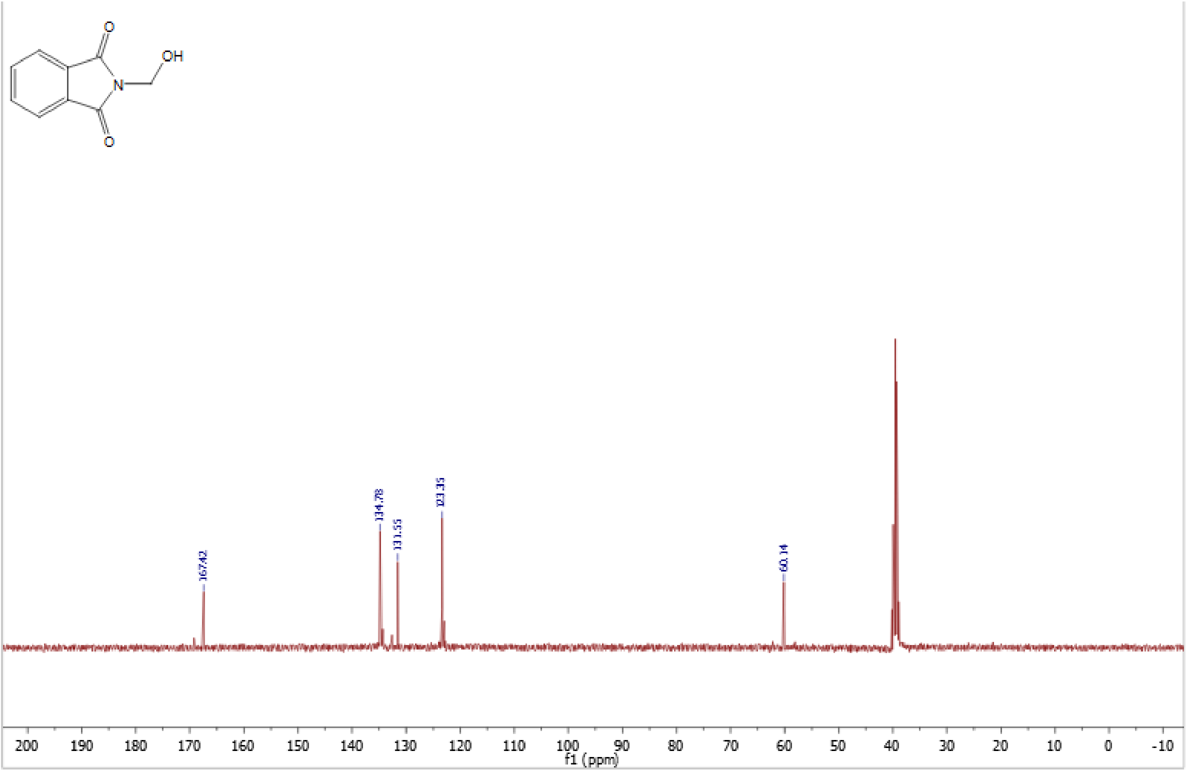

NMR spectra of 1,3,3-Trimethyl-2-methylene-5-(phthalimidomethyl)-indolenine

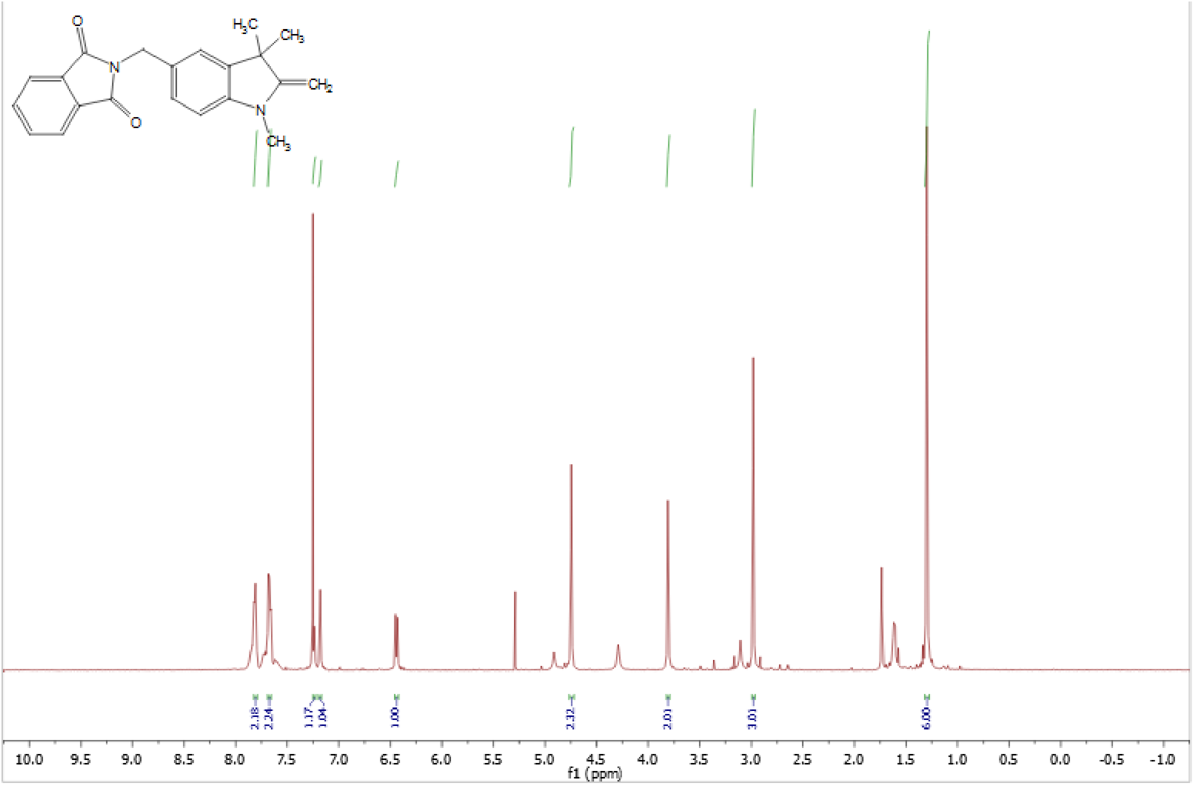

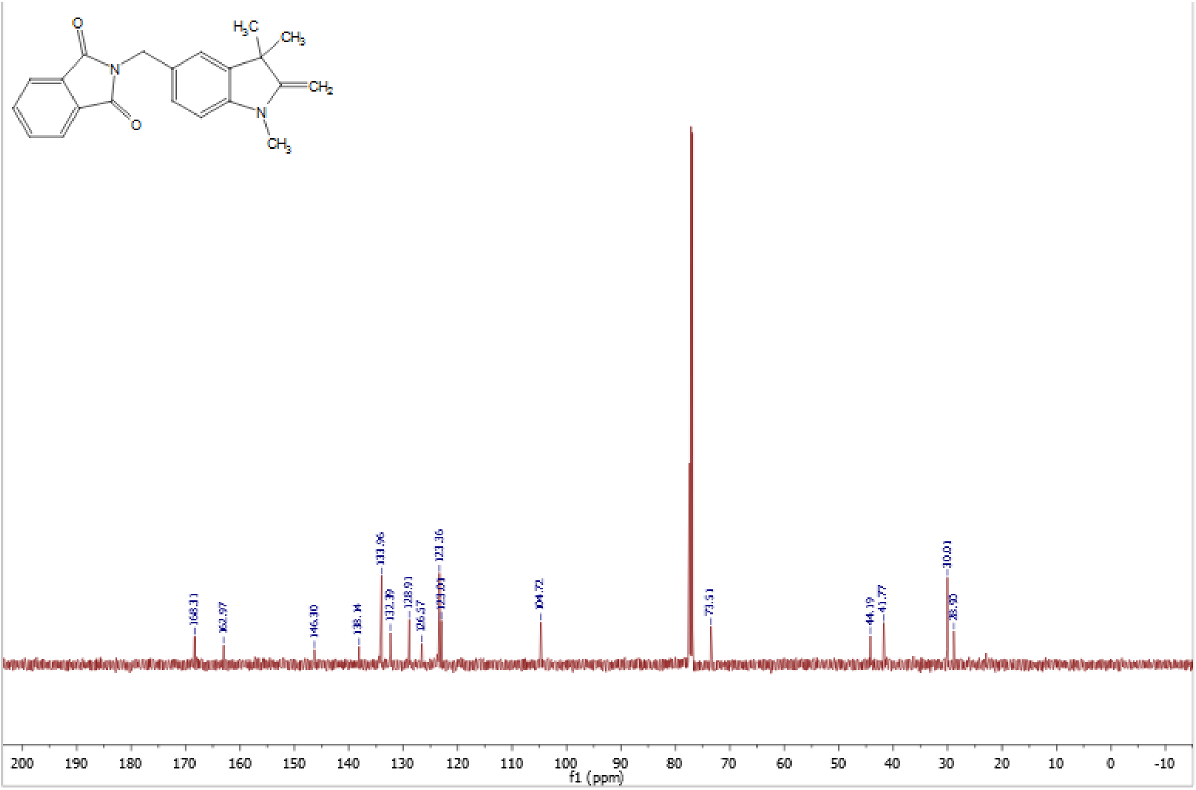

NMR spectra of ICC dye **1**

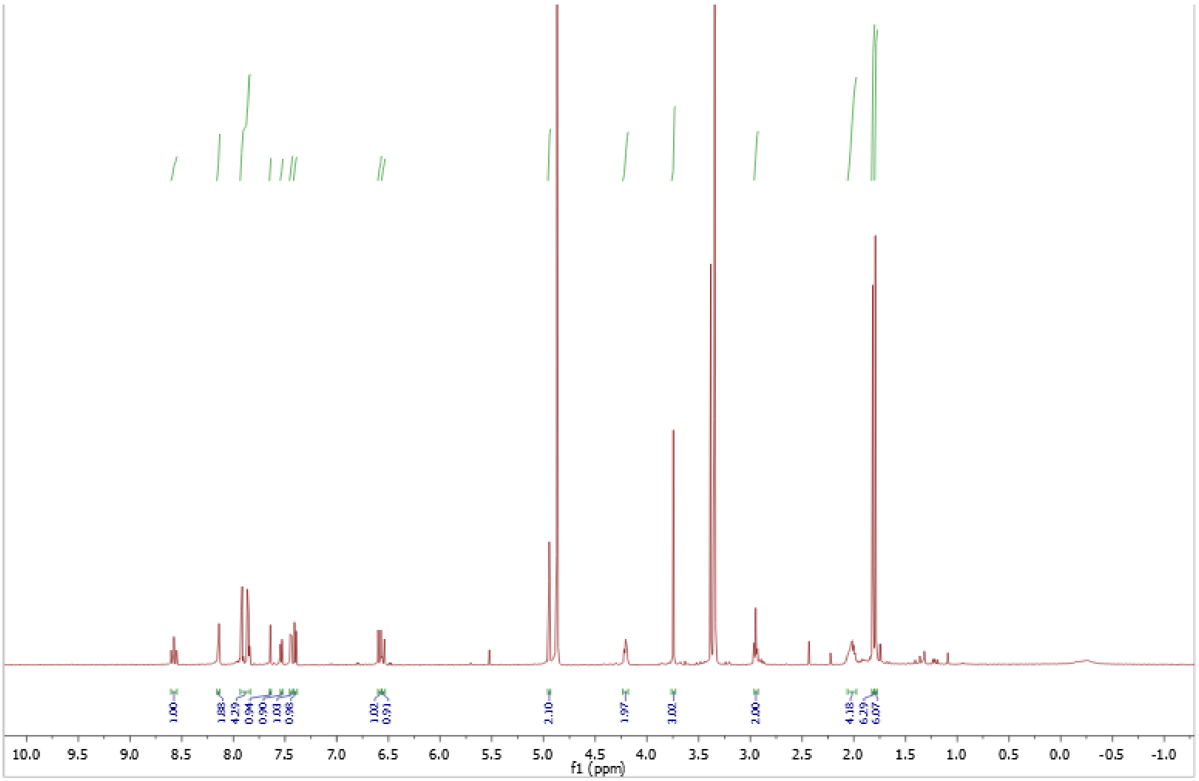

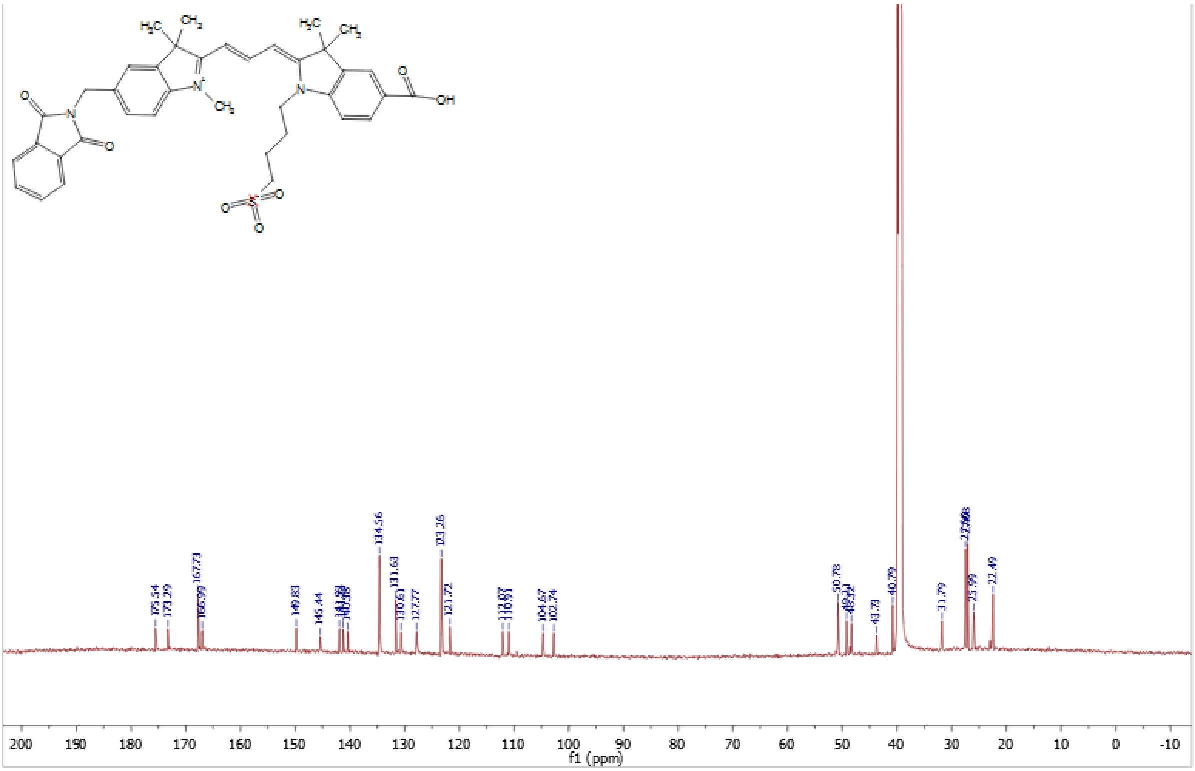

NMR spectra of ICC dye **2**

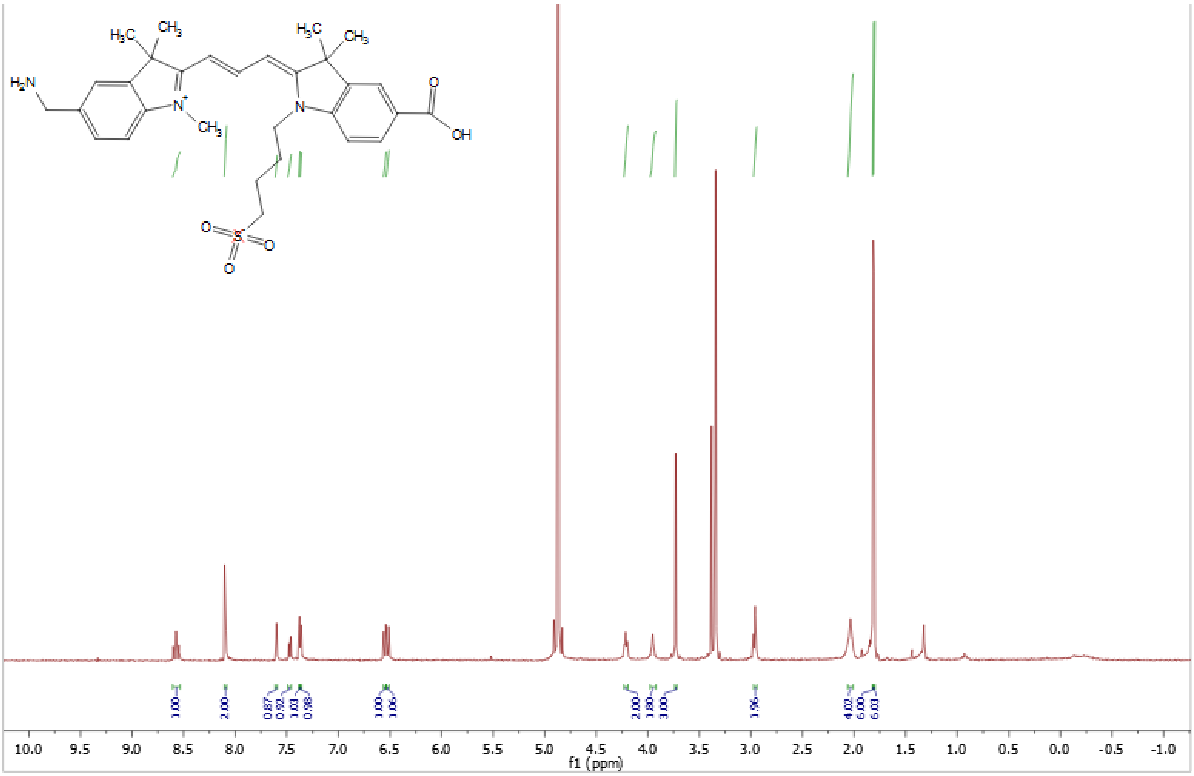

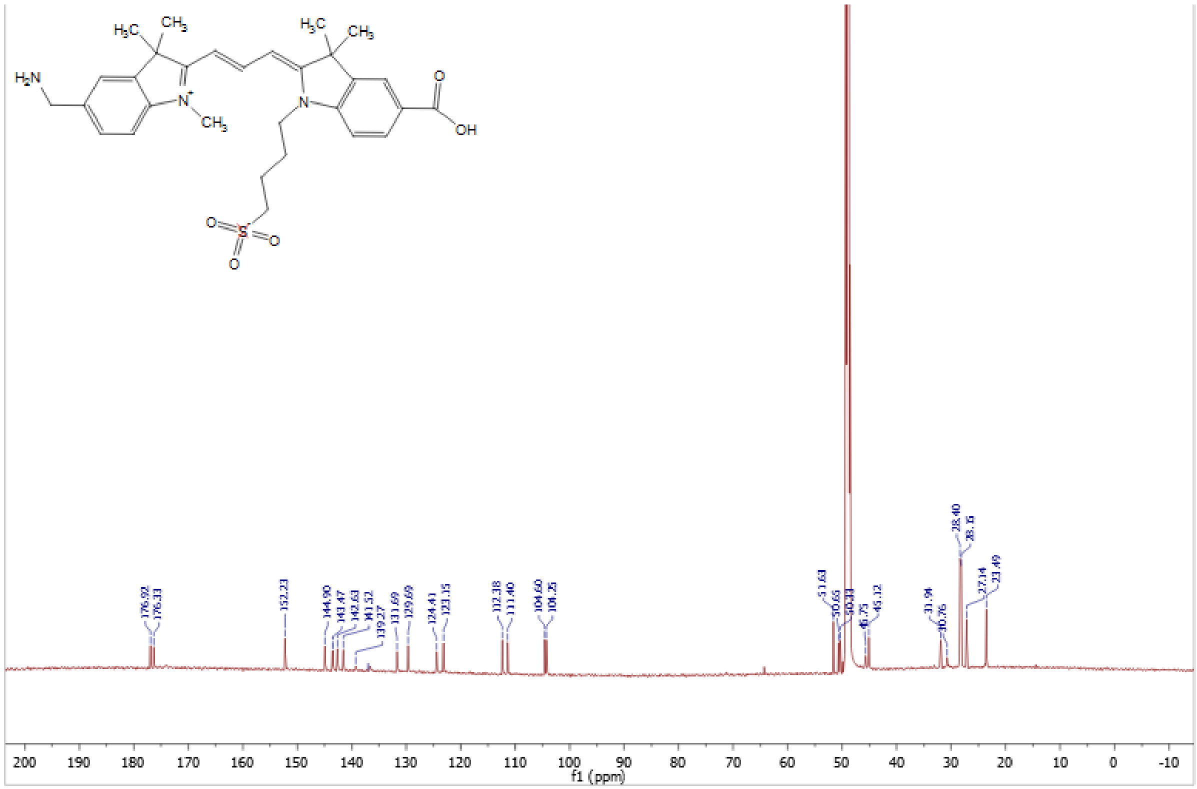

NMR spectra of DOTA-ICC label **3**

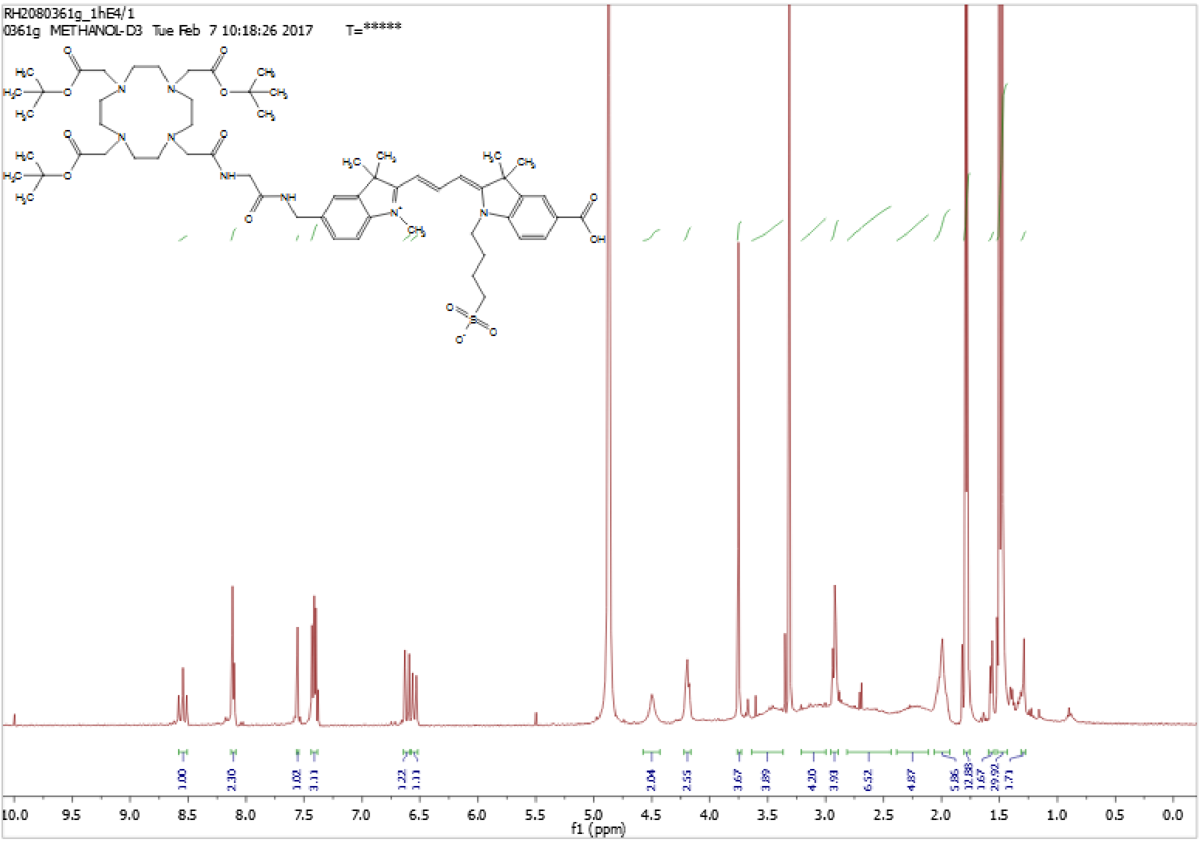

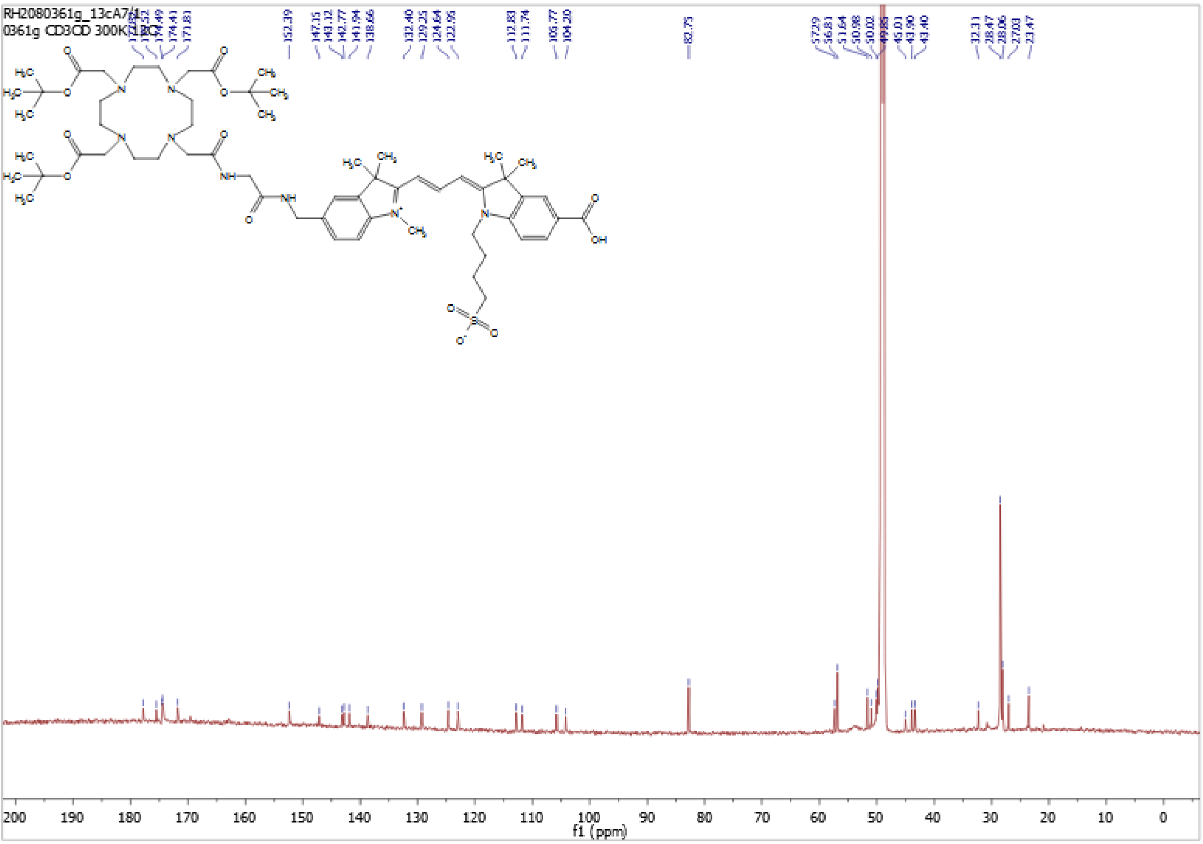

NMR spectra of ^{nat}Ga-DOTA-ICC (including ^{71}Ga NMR)

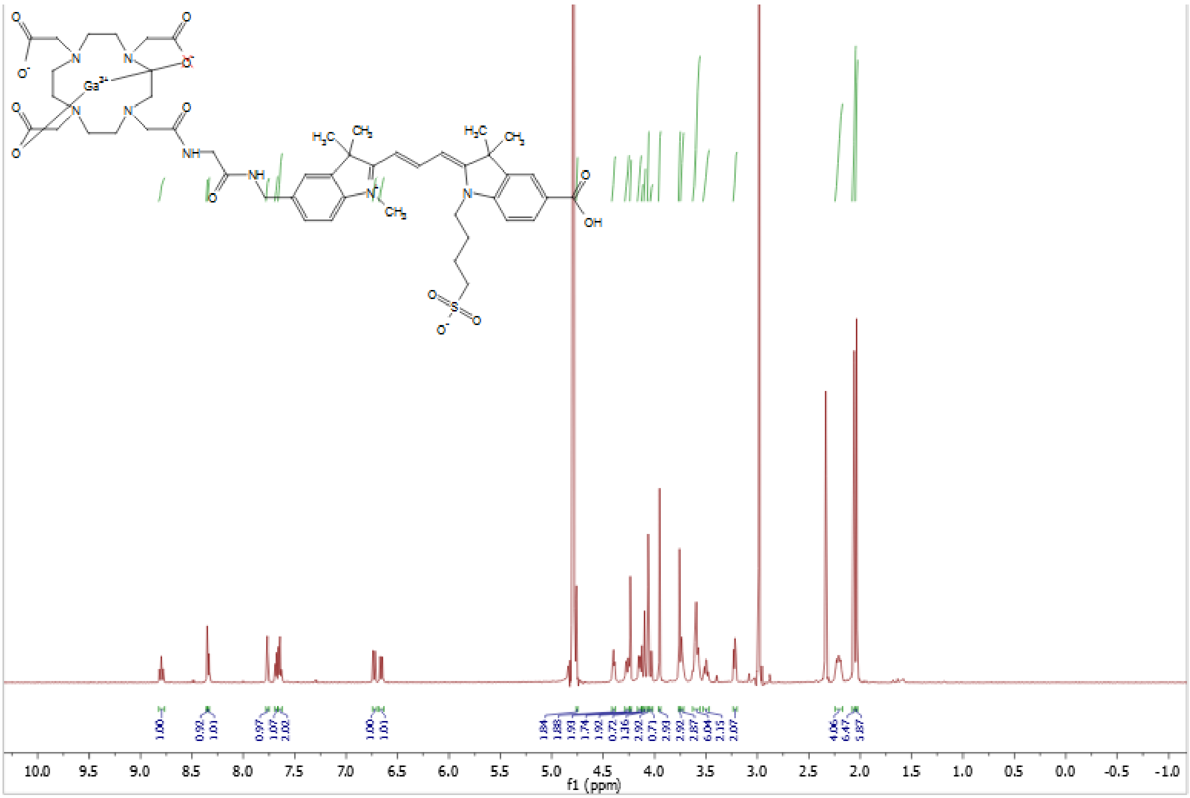

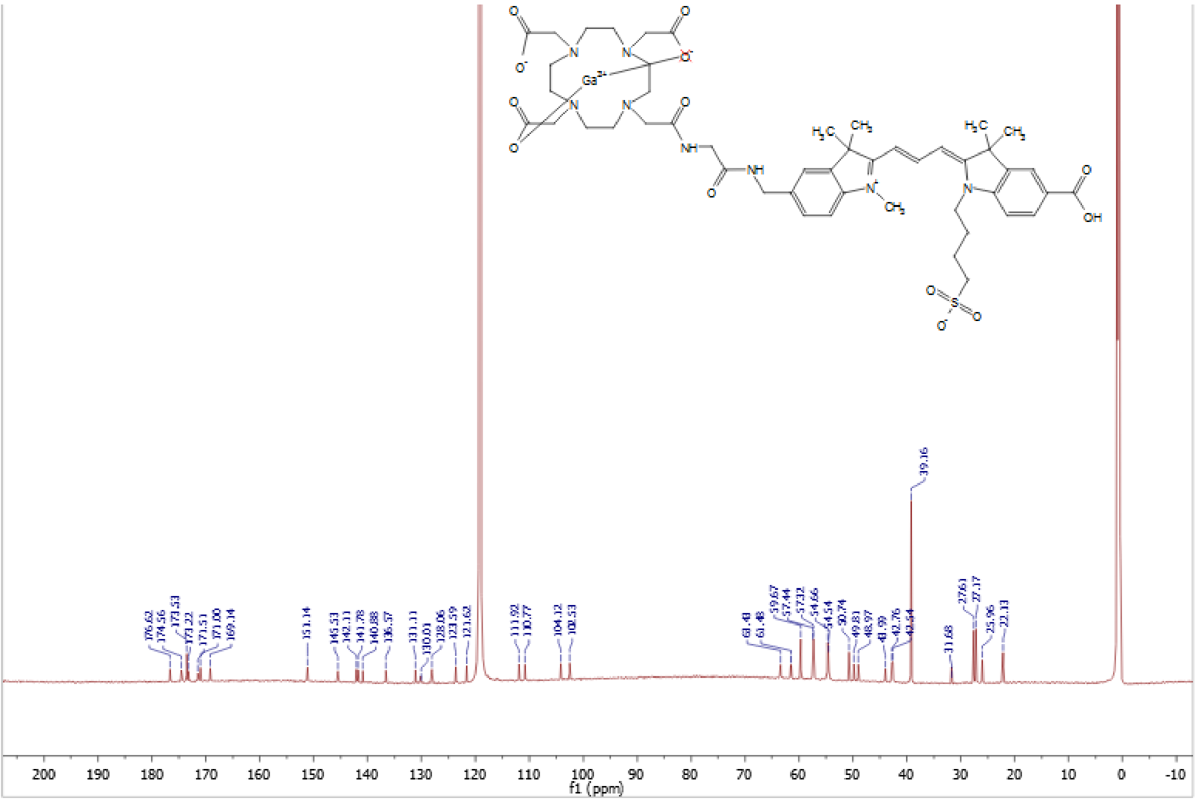

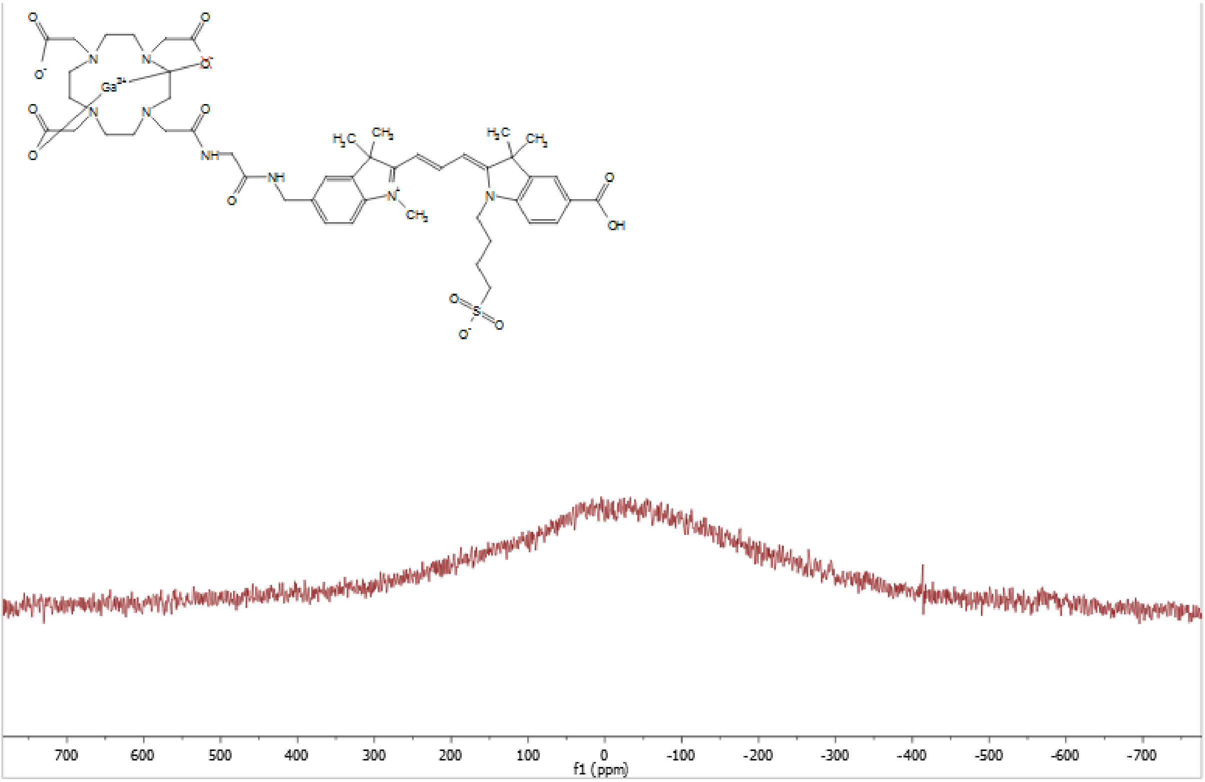

Ga3+
NH
NH
OH
f1 (ppm)
700
600
500
400
300
200
100
0
-100
-200
-300
-400
-500
-600
-700

5. Mass spectra

Mass spectrum of *N*-(Hydroxymethyl)-phthalimide

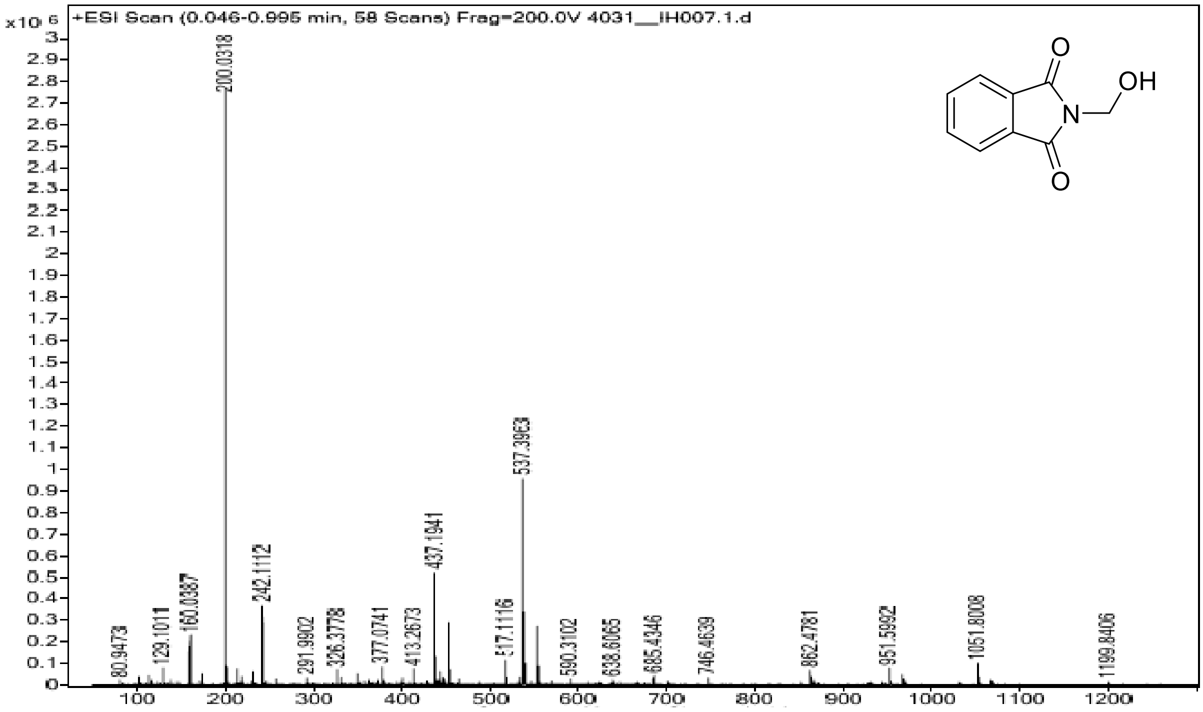

Mass spectrum of 1,3,3-Trimethyl-2-methylene-5-(phthalimidomethyl)-indolenine

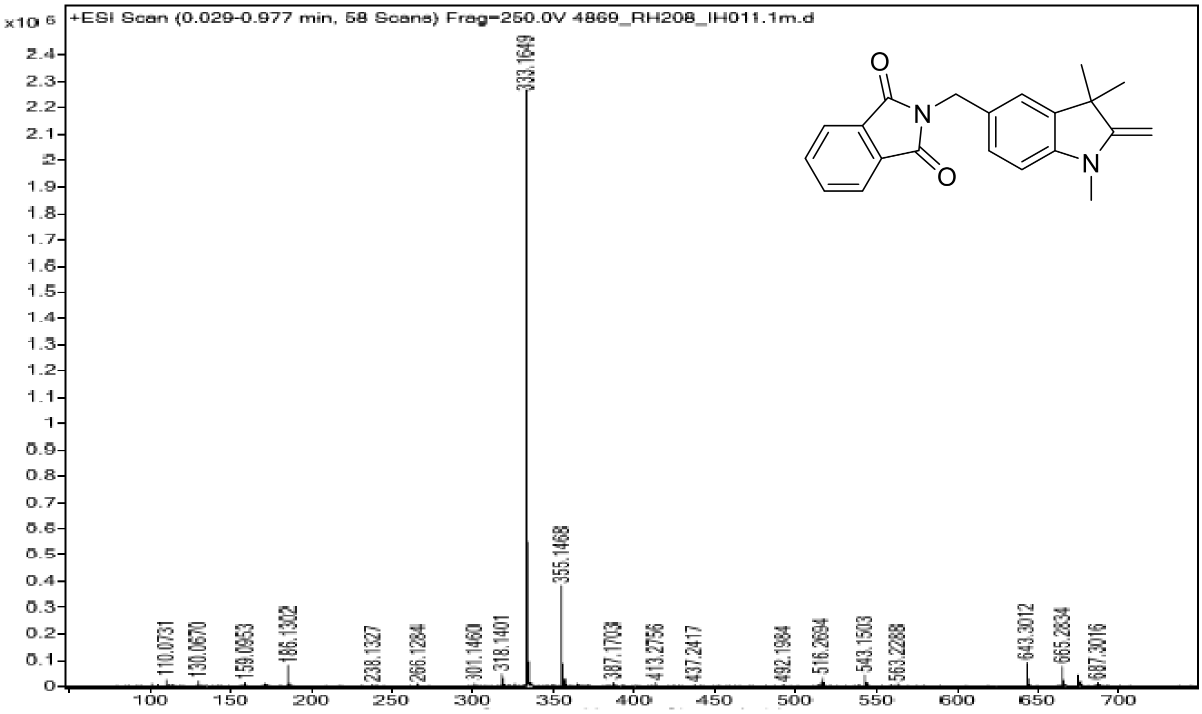

Mass spectrum of ICC dye **1**

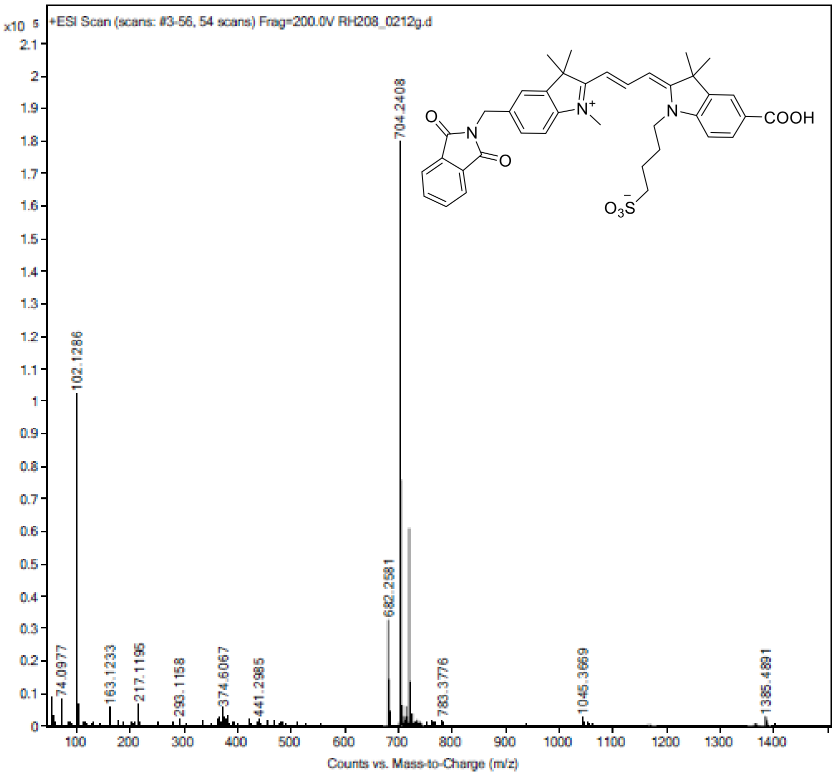

Mass spectrum of ICC dye **2**

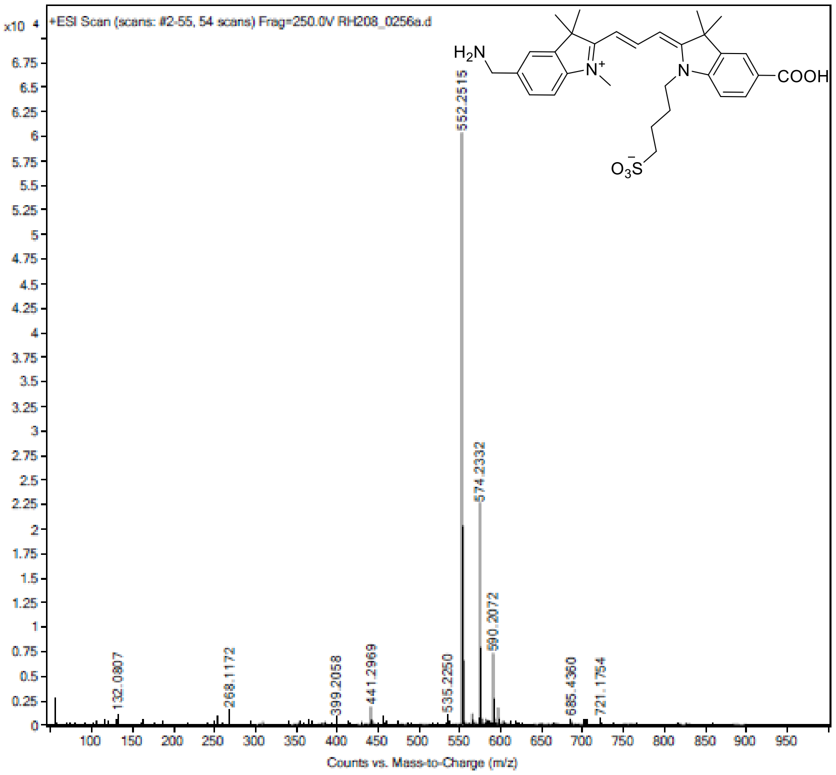

Mass spectrum of DOTA-ICC label **3**

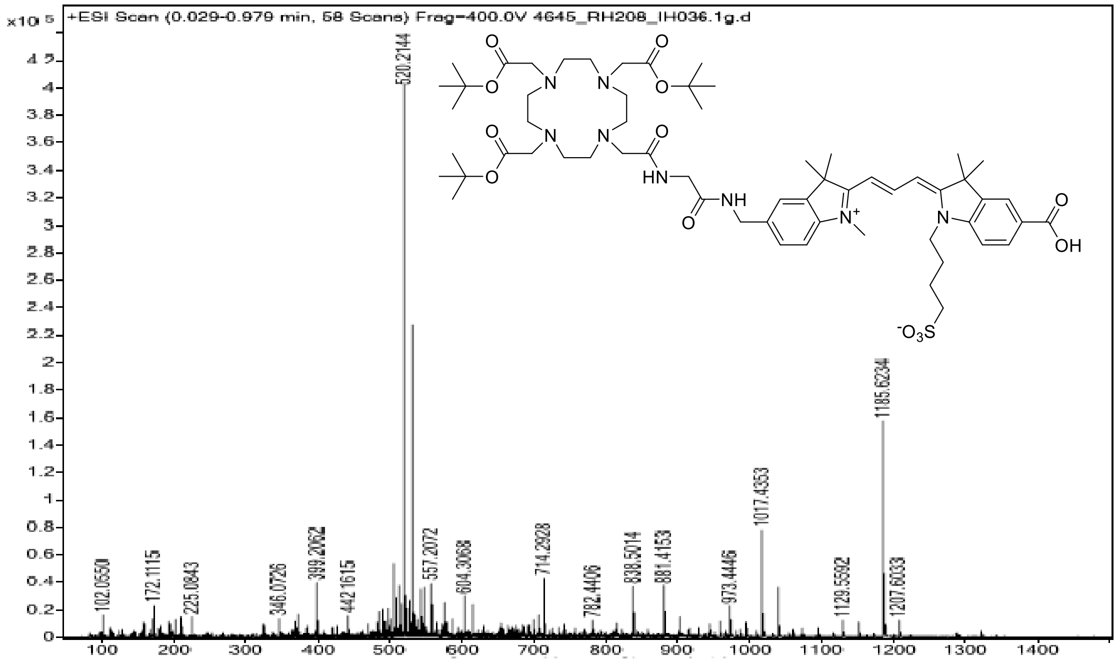

Mass spectra of ^{nat}Ga-DOTA-ICC

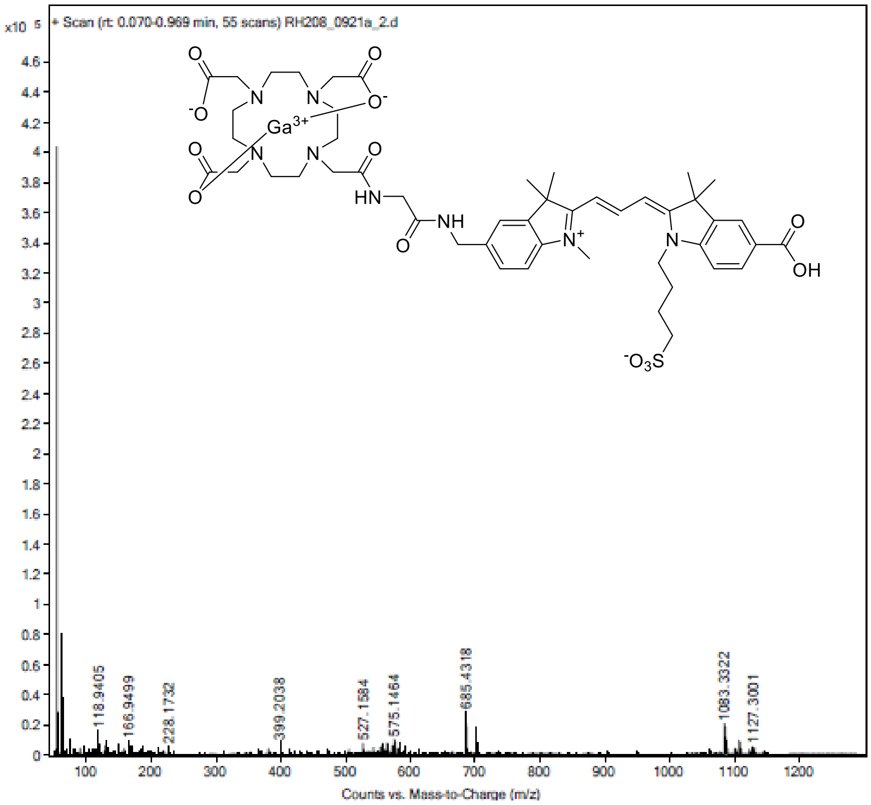

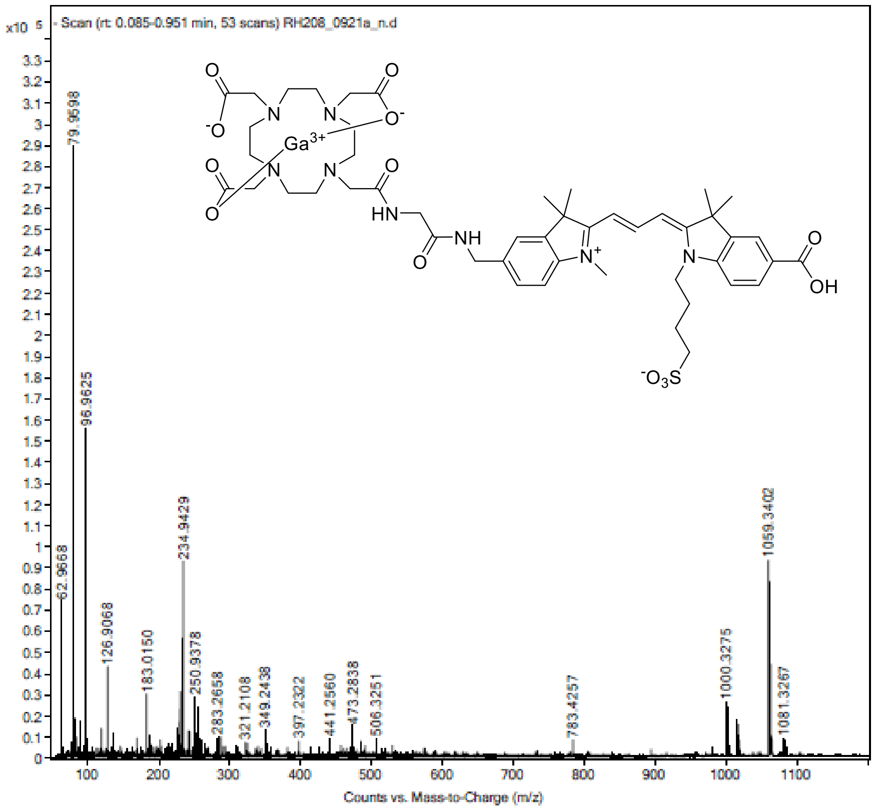

Mass spectrum of the deprotected DOTA-ICC-TATE conjugate

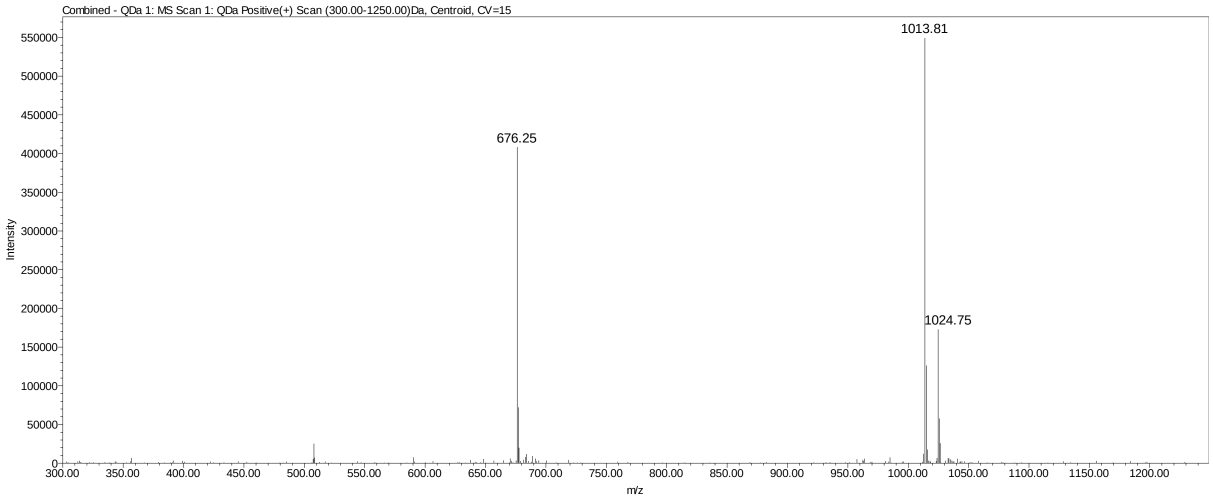

6. Chromatograms

Chromatogram of the deprotected DOTA-ICC-TATE conjugate

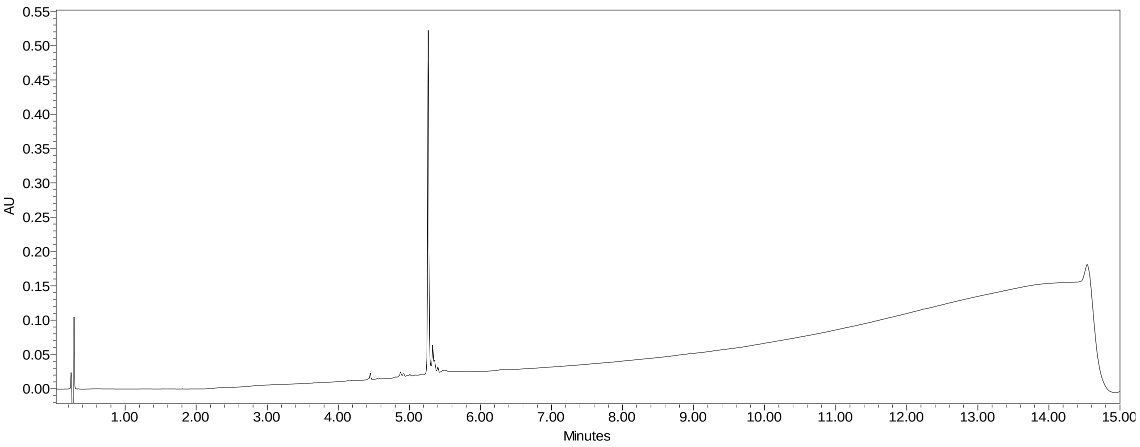

Chromatograms of the DOTA-ICC-TATE (red, t_R = 12.8 min) and ^{nat}Ga-DOTA-ICC-TATE (blue, t_R = 13.4 min) conjugates

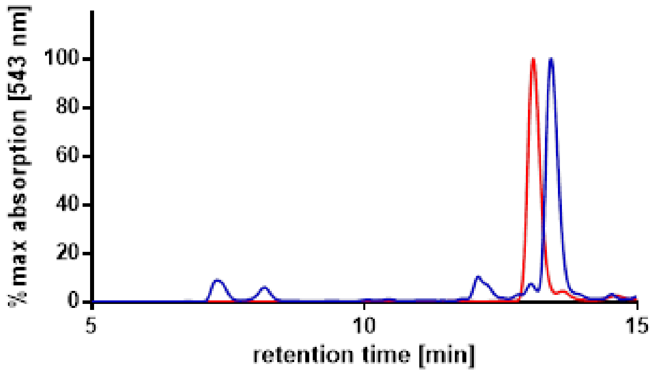

Chromatogram of the ^{68}Ga-DOTA-ICC-TATE conjugate

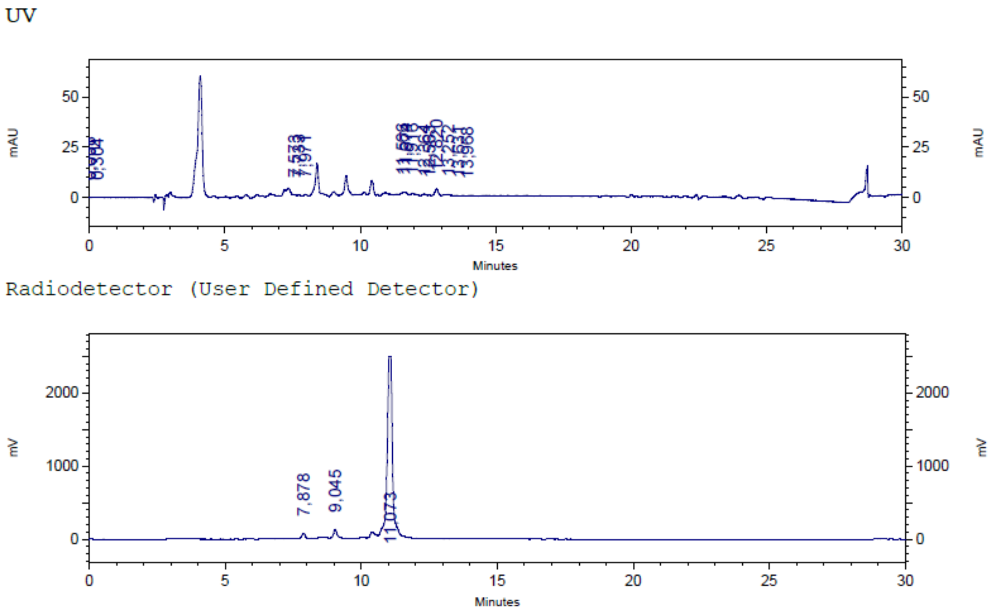

Supplement: Supplementary file 1 — Supplementary [file CBIC-22-1307-s001.pdf]
